# Analysis and Improvements in AprilTag Based State Estimation

**DOI:** 10.3390/s19245480

**Published:** 2019-12-12

**Authors:** Syed Muhammad Abbas, Salman Aslam, Karsten Berns, Abubakr Muhammad

**Affiliations:** 1 Department of Electrical Engineering, Lahore University of Management Sciences (LUMS), Lahore 54792, Pakistan; salman@gatech.edu (S.A.); abubakr@lums.edu.pk (A.M.); 2 Department of Computer Science, University of Kaiserslautern, D-67663 Kaiserslautern, Germany; berns@informatik.uni-kl.de

**Keywords:** robot sensing and perception, sensor modelling, localization

## Abstract

In this paper, we analyzed the accuracy and precision of AprilTag as a visual fiducial marker in detail. We have analyzed error propagation along two horizontal axes along with the effect of angular rotation about the vertical axis. We have identified that the angular rotation of the camera (yaw angle) about its vertical axis is the primary source of error that decreases the precision to the point where the marker system is not potentially viable for sub-decimeter precise tasks. Other factors are the distance and viewing angle of the camera from the AprilTag. Based on these observations, three improvement steps have been proposed. One is the trigonometric correction of the yaw angle to point the camera towards the center of the tag. Second, the use of a custom-built yaw-axis gimbal, which tracks the center of the tag in real-time. Third, we have presented for the first time a pose-indexed probabilistic sensor error model of the AprilTag using a Gaussian Processes based regression of experimental data, validated by particle filter tracking. Our proposed approach, which can be deployed with all three improvement steps, increases the system’s overall accuracy and precision by manifolds with a slight trade-off with execution time over commonly available AprilTag library. These proposed improvements make AprilTag suitable to be used as precision localization systems for outdoor and indoor applications.

## 1. Introduction

Localization capability is the backbone of many robotic systems as it helps determine the state of the robot at a given time instance [1]. Many essential subsystems of an autonomous mobile system take localization as an input for developing maps or plan navigation strategies [2]. The nature of the application determines the level of localization accuracy required. Localization accuracy is commonly measured by comparing it with the ground truth at any given time instance. Therefore, the ground truth itself must be of superior accuracy to minimize the error in measuring localization accuracy. In robotics, there exist several ways of generating ground truth. A standard method for generating ground truth for localization is with the help of high precision motion caption cameras (MoCap) [3]. This system is considered to be the benchmark for many indoor localization systems worldwide. MoCap setup contains multiple cameras calibrated at known positions, fuse the data to track a known marker at high accuracy. For indoor applications, another commonly used method is the use of fiducial or visual marker-based localization systems. This method is quite popular because of the readiness to use it. For many applications, fiducial markers’ relative ease of use makes them the primary method for localization [4]. Besides being used for outdoor applications, GPS is the most popular source for ground truth verification [5]. Although it is globally consistent, nominal GPS systems do not provide adequate accuracy for tasks that demand sub-meter localization accuracies such as robot navigation, obstacle avoidance or structural inspection in confined environments. Some high-end GPS methodologies such as D-GPS and RKT-GPS have an accuracy of 0.1 meters or less but they are quite expensive and are hard to setup. In outdoor environments, the deployment of fiducial marker-based systems are also possible but they have limitations on operating distance and field-of-view.

AprilTag is one of the most commonly used fiducial markers that can be used both indoors and outdoors for ground truth generation in 6-DOF, but with limitations [6]. We have precisely identified these limitations and have explained the source of these limitations with statistical error models. The proposed research has established that both distance and orientation of viewing camera from the target tag effects accuracy. However, uncorrected orientation uncertainty is a more significant source of accuracy degradation. AprilTag’s accuracy is maximum when the viewing camera is pointed towards the center of the tag. Moreover, in the current implementation of the AprilTag localization system, this source of error is left unaddressed. As a result, the system suffers from a loss of performance, which is rectifiable. The proposed research has filled this gap (only for 2D) via an empirical analysis of the AprilTag system. Furthermore, a data-driven probabilistic sensor model has also been proposed, which works both in indoor and outdoor environments.

In this paper, we have proposed techniques to overcome this limitation and to increase the accuracy even for wider horizontal viewing angles. The proposed technique consists of three approaches. One is a geometric *soft correction* to the displacement angle from the center of the tag. Second is a *active correction* to the angular displacement using a custom-built gimbal which detects the tag in real-time and physically keeps the camera viewing angle towards the center of the AprilTag horizontally. The third is a proposal of a probabilistic sensor error model of the AprilTag by using Gaussian Processes (GP) based regression of experimental data. The forward sensor model is directly usable in a standard Baye’s filter for localization, mapping, SLAM or exploration algorithms [2]. We have used these approaches in combination and a detailed comparison is also presented to determine how different approaches have improved the overall precision. For example, in an ideal scenario, we have improved the accuracy from 4.4 cm to 0.8 cm in the x-axis and 2.56 cm to 0.54 cm in the y-axis. Moreover, other than the accuracy, improvement in the precision has also been achieved from 112 cm${}^{2}$ to 0.29 cm${}^{2}$ for the x-axis and 14 cm${}^{2}$ to 0.60 cm${}^{2}$ for the y-axis over a target distance of 70 cm. All the AprilTag measurements used in data error comparisons are raw and without modifications.

In Section 2, an overview of the related work on visual marker systems has been discussed. This section describes different fiducial markers, their techniques and their applications. Section 3 illustrates the problem set up and the evaluation of AprilTag as a localization system. In this section, the implementation methodology of AprilTag is briefly discussed, then details regarding transformations required for trajectory generation is discussed. Then the error measurement setup is explained along with the method for taking measurements and lately identification of the AprilTag’s shortcomings. Section 4 discusses the reasons behind the identified shortcomings and proposes improvement techniques. Lately, a detailed comparison of all the proposed improvement approaches is presented. Afterward, a probabilistic sensor model for AprilTag has been proposed by using the Gaussian Processes (GP) regression along with the experimental verification of the proposed sensor model by implementing trajectory tracking using particle filter both in a laboratory setup and in an outdoor environment. Lastly, Section 5 concludes the whole paper with the identification of future work required.

## 2. Related Work

A visual fiducial system uses 2D coded information embedded on a tag to give the position and the orientation of the marker to the camera. The 2D coded information also distinguishes between one marker from the other. Distinct fiducial systems are being used in robotics applications for pose estimation. All are best known for their use in augmented reality applications to support vision-based tracking [7]. Table 1 shows an overview of different commonly used fiducial markers in robotics application along with their key features.

In 2011, Olson [6] showed that AprilTag surpasses its predecessors in terms of detection rate, inter-coding Hamming distance, scale and angular-accuracy. Olson has also addressed the accuracy issues of AprilTag related to tag-detection percentage along with the camera distance from the tag. However, the results are not extensive enough to use them in creating a perfect sensor model for AprilTag and missed some necessary details which follow in the next sections. Nonetheless, because of more robustness and accuracy of AprilTag [6], many researchers have preferred it over any other visual fiducial markers so far. In 2017, Sagitov et al. [16] compared ARTag, AprilTag and CALTag for occlusions and showed that AprilTag is robust against small occlusions.

One of the advantages of AprilTag is the utilization as a low-cost localization solution in augmented reality and robotics applications. The setup requires only a monocular camera and a printed AprilTag on a paper. As a result, researchers prefer fiducial markers over other high-end localization systems. C Feng et al. [17] have used AprilTag as spatial indices for operations like navigation and inspection inside a building for engineering, construction and management related tasks. They have placed AprilTags on different parts of the building, which direct users with operation-specific information when seen through a mobile camera. Li et al. [18] combine the fiducial marker with inertial sensors to have an improved position and pose tracking of hand-held augmented reality system. They achieved an accuracy of 1.77 cm for the position and $4.15^{\circ }$ for orientation estimation. Some researchers have used AprilTag as a landmark and track it in robotics applications. Wang et al. [19] and Wang [20] have proposed a vision-based vehicle tracking system in which an unmanned aerial vehicle (UAV) tracks a ground vehicle by using AprilTag attached to a ground vehicle. Ling et al. [21] have used AprilTag attached over a water vessel, to autonomously land an unmanned aerial vehicle (UAV) over it. Similarly, Zhang et al. [22] have used an identical approach to land the aerial vehicle over land using AprilTag. Later, a similar work regarding the autonomous landing of a quadrotor using AprilTag is done by Reference [23]. Tang et al. [24] have proposed an algorithm to fuse the data from multiple cameras and a 2D laser scanner. They have used an array of AprilTags as a target for calibration and employ a non-linear optimization technique to estimate a single camera intrinsic parameters out of multiple cameras and later fuse them with 2D laser scanner data to have an improved position and pose estimate.

Another advantage of AprilTag is for the accurate evaluation of individual localization systems or algorithms. Ramirez [25] has made a dataset for visual odometry and localization in which he used AprilTags as landmarks for accuracy evaluation. Parkison et al. [26] have used AprilTag to evaluate the position and pose of a micro aerial vehicle (MAV) for automated indoor RFID inventorying. Raina et al. [27] have used multiple AprilTags as a ground truth evaluation system for 3D pose estimation in a cluttered environment. Maragh [28] has used AprilTag to control the position and angular velocity of a rotating body using PD control. She has also demonstrated the upper limit of the angular velocity of a moving object for the robust detection of AprilTag. Similarly, Zake et al. [29] have used AprilTag measurements as a ground-truth value to compare the output of the proposed pose-based visual serving technique for cable-driven robots. Florea et al. [30] have used AprilTags to localize a drone and other multiple waypoints as they have proposed a sensor fusion technique for localization by using numerical P systems. Researchers have also used AprilTags for modeling the dynamics of different physical systems. Britto et al. [31] have used fiducial marker AprilTag to estimate the position and orientation of an unmanned underwater vehicle (UUV), later used in the dynamic model of the underwater system. Fuchs et al. [32] have used AprilTag for the kinematic modeling and trajectory generation of a trailer attached to a truck using Kalman Filter [33]. Nissler et al. have used AprilTag for the robot to camera calibration to get the exact pose of each robot part in the camera frame of reference for precise operations. Mueggler et al. [34] have used multiple AprilTags to precisely estimated the position of an aerial vehicle in a swarm rescue operation. They have successfully demonstrated in a laboratory setup because localization from AprilTags has played an integral part in the completion of the task. Xie et al. [35] have used AprilTag to find the pose and extrinsic of multi-camera and multi-LiDAR system. They have shown that by using AprilTags in calibration process improves the overall robustness and accuracy of an autonomous driving platform. Later Nissler et al. [36] have used single and multiple AprilTags with a high-end camera to estimate the position and orientation of a manipulator in an industrial environment. They have shown that the use of AprilTag can help increase the precision tasks of a manipulator. Similarly, De et al. [37] have used AprilTag for the pose estimation in visual-inertial navigation of a real-time MAV application in an indoor environment.

The disadvantage of using AprilTag as a localization system may result in erroneous localization due to multiple factors. These factors include configurations such as viewing angle, distance and camera rotation around its axis. Though AprilTag has been used in many applications ranging from virtual reality to tracking and localization, there are not many studies related to a systematic analysis of how the inaccuracy propagates over different distances and viewing angles. A similar study regarding the accurate evaluation of a similar fiducial marker (ARToolKit) has been conducted by Abawi et al. [38]. They have experimentally calculated the accuracy of ARToolKit, which is a similar fiducial marker as AprilTag but far less robust and accurate, as demonstrated by Reference [6]. They have given a conclusion that ARToolKit is accurate for short distances and for viewing angle between $40^{\circ }$ and $80^{\circ }$. Furthermore, Wang et al. [39] have also proposed improvements in AprilTag but those improvements are limited to improving tag detection and lowering computational utilization and called it Apriltag 2. In 2017, Jin et al. [40] showed that the AprilTag pose output is inaccurate and noisy. They have proposed that by adding depth information along with the RGB information of the tag improves the overall pose accuracy. They have used an RGB-D camera to detect an AprilTag in an indoor setup. However, the proposed method fails outdoors as the RGBD camera does not work outdoors in direct sunlight. Zhenglong et al. [41] have used multiple AprilTags to estimate the pose of a flying quadrotor better. They have experimented in an indoor environment by laying multiple AprilTags on the floor and have flown a multirotor with a down-looking camera. A Kalman Filter with a constant velocity model has been used to estimate a more accurate pose by fusing poses from multiple AprilTags. It is shown that it has improved the overall pose estimation and matched it with a Motion Capture (MoCap). However, for a large outdoor environment, it is not possible to lay multiple AprilTags on the ground beneath a flying robot all the time for pose correction, so this makes the proposed approach not suitable for a large outdoor environment. In 2019, Kayhani et al. [42] have proposed that the raw AprilTag pose is not accurate enough for autonomous operations and has improved the accuracy of an indoor multi-copter by fusing pose data from multiple AprilTags with the help of an Extended Kalman Filter.

Some researchers have evaluated the pose accuracy of the AprilTag in indoor environments and have improved it by using multiple tags along with data fusion techniques such as Kalman Filtering. Though they have improved the detected pose accuracy by fusing data from multiple tags, their proposed setup can only be possible in small indoor environments. For a large outdoor environment, it is still an open question. Moreover, AprilTags can be used for ground-truth analysis in many autonomous applications such as self-driving cars [43]. Hence, improving AprilTag accuracy to the point where it serves as a ground-truth solution, especially outdoors, is still an open challenge.

## 3. Problem Setup and System Evaluation

### 3.1. AprilTag Working Principle

**Figure 1:**
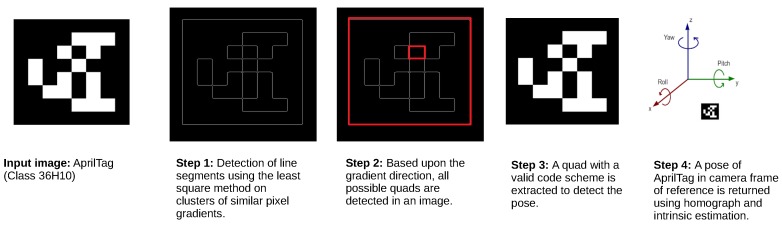
Figure shows four steps of AprilTag detection algorithm with an input image of AprilTag of class 36H10.

As described in the earlier section, AprilTag also uses an embedded 2D-coded marker for tag detection and to differentiate it from the other tags. The visual marker tag can be of any size with a square dimension. The tag is printed on a white background with a black outline square. Inside the square is an embedded black bar-code. AprilTag [6] uses a unique detection algorithm for fast, robust detection and to minimize the effect of small occlusions. Figure 1 shows the algorithmic steps of AprilTag. In the first step, it computes the magnitude and direction of a gradient at every pixel in an image that contains the AprilTag. Afterward, these calculated gradients are grouped into clusters called components based on similar gradient attributes using a graph-based method. By using a weighted least square technique, a line is fitted on every component such that the direction of the gradients determines the direction of the fitted line. Moreover, gradient direction determines the direction of the line segments. Hence each line has a dark side on its left and a lighter side on its right. Furthermore, after identifying all lines, possible quad shapes are detected, as shown in step 3 of Figure 1. The quad shape with a valid code scheme is extracted out. Also, a 6-DOF pose of the tag in the camera frame of reference is returned by using homography and intrinsic estimation over an extracted tag.

### 3.2. Trajectory Generation

**Figure 2:**
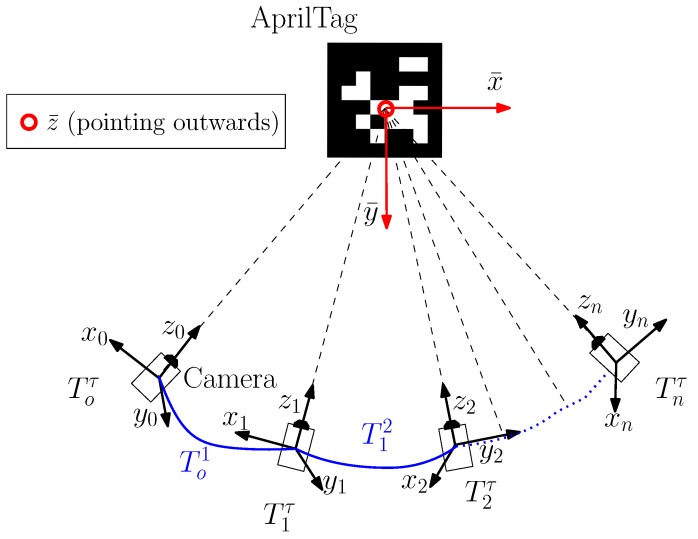
Trajectory using AprilTag detections. The trail of the transformation frame centers that constitute the trajectory is depicted in blue for various time instances. Here, $p_{i_{x}}$,$p_{i_{y}}$ and $p_{i_{z}}$ of Equation (1) (although not shown in the figure) depict the position of AprilTag in $x_{i}$-axis, $y_{i}$-axis and $z_{i}$-axis in the respective camera frame of reference.

In all robotics applications, odometry is key to every operation. Odometry includes all the positions and poses of a moving robot along its timestamp. As discussed in the literature survey, AprilTag is widely used for odometry generation of robots both in indoor and outdoor applications. It is illustrated in the previous section, AprilTag returns a single pose in 6-DOF relative to the camera frame of reference. Moreover, as the camera mounted on a robot changes its position along with the motion of a robot, it produces a series of posses from AprilTag at each time instance. Each pose shows the position of a robot along the moving robot trajectory at a particular time instance. Furthermore, to have a continuous trajectory we proposed a standard transformation technique between any two consecutive poses as shown in Figure 2. Suppose, we get a 6-DOF pose from an AprilTag in camera frame of reference, hence the camera attached frame, described in the Tag frame of reference $\tau =(\bar{x},\bar{y},\bar{z})$ at instance *i*, is given by the homogeneous transformation:   

(1) $$\begin{array}{c} T_{i}^{\tau }={\left[\begin{array}{cc} R_{i} & d_{i}\\ 0 & 1 \end{array}\right]}_{4\times 4},\;d_{i}={\left[\begin{array}{ccc} p_{i_{x}} & p_{i_{y}} & p_{i_{z}} \end{array}\right]}^{T}\\ R_{i}=\left[\begin{array}{ccc} c\phi c\theta & c\theta s\psi s\phi -c\psi s\theta & c\psi c\theta s\phi +s\psi s\theta \\ c\phi s\theta & c\psi c\theta +s\psi s\phi s\theta & -c\theta s\psi +c\psi s\phi s\theta \\ -s\phi & c\phi s\psi & c\psi c\phi \end{array}\right]. \end{array}$$

In Equation (1), the input angles are in camera frame of reference such as ‘θ’ is the rotation about $z_{i}$-axis and represents the roll motion of the camera, ‘ϕ’ is the rotation about $y_{i}$-axis and represents the yaw motion of the camera and ‘ψ’ is the rotation about $x_{i}$-axis and represents the pitch motion of the camera where i=0,1,2,...n. $p_{i_{x}},p_{i_{y}},p_{i_{z}}$ are the displacements in x-axis, y-axis and z-axis respectively in camera frame of reference. To have a trajectory in a single frame of reference, we need to find transformation $T_{i}^{i+1}$ from point $p_{i}$ to $p_{i+1}$.

(2) $$T_{i}^{i+1}=T_{\tau }^{i+1}\times {\left(T_{\tau }^{i}\right)}^{-1},\mathrm{where}{\left(T\right)}^{-1}=\left[\begin{array}{cc} R^{T} & -R^{T}d\\ 0 & 1 \end{array}\right].$$

In practice, AprilTag is detected at 10 HZ and the trajectory becomes almost continuous due to slow camera movement.

### 3.3. Error Measurements Setup

**Figure 3:**
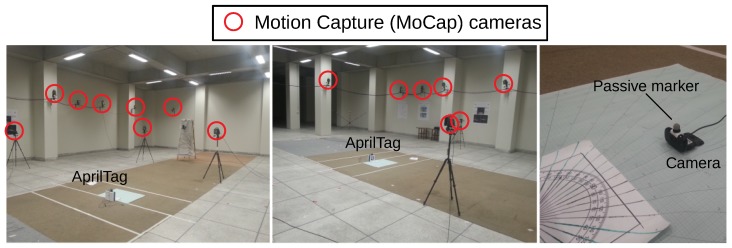
Motion Capture (MoCap) setup at LUMS Biomechanics lab for AprilTag comparison.

To analysis, the accuracy and precision of AprilTag, raw readings from AprilTag’s native implementation have been compared with the readings of a high precision localization system called ViconMX−F40, also known as “Motion Caption (MoCap)” [3]. MoCap consists of 16 high frame-rate cameras placed at the different known positions in an indoor environment. The system optically tracks a passive marker in 6-DOF with the sub-centimeter precision. It also has the capability of tracking multiple passive markers. This system is considered as a benchmark for all indoor localization problems. Multiple monocular cameras which are calibrated at known positions, fuse the optical tracking data of a marker to track at good accuracy as shown in Figure 3.

AprilTag technology requires accuracy to the centimeter for the analysis, which is why we used a Motion Capture System (MoCap) for ground truth measurements. As described earlier, MoCap is an optical system that detects a passive marker; hence, a passive marker has been mounted above the camera to detect the position and orientation of the camera. For AprilTag localization, the measurement process has been made simple by making the origins of both AprilTag and MoCap frame of references aligned. Also, the camera is placed over a robotic platform that moves randomly around and the mounted camera has a constant motion of maximum $30^{\circ }$ around its yaw axis to include maximum noise possible at a given nominal reference point. Table 2 shows the overall performance of the MoCap for estimating the robot’s position on the ground. Column 1 ‘$x_{r}$’ and column 2 ‘$y_{r}$’ in Table 2 show the nominal positions of reference points from which the measurements have been taken. Column 3 ‘*N*’ represents the total number of readings taken at a specific reference point, column 4 ‘$\mu_{\bar{x}}$’ and column 5 ‘$\mu_{\bar{y}}$’ shows the accuracy of the MoCap as the reported mean value in both *x*-axis and *y*-axis respectively. Lastly, column 6 ‘$\sigma_{\bar{x}}^{2}$’ and column 7 ‘$\sigma_{\bar{y}}^{2}$’ shows the precision of the MoCap in the form of variances reported in *x*-axis and *y*-axis respectively.

**Figure 4:**
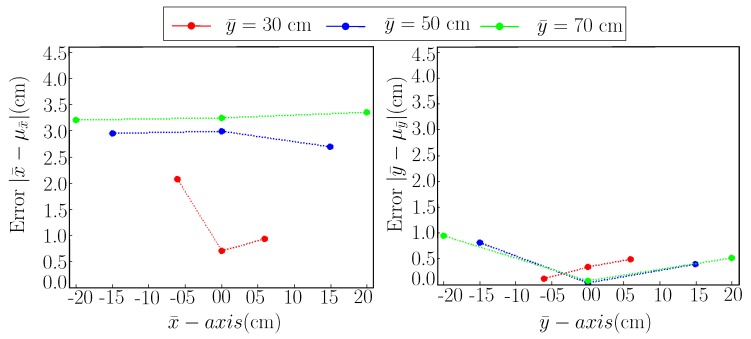
Accuracy plot for Motion Capture (MoCap).

Moreover, Figure 4 shows the error plot for both $\bar{x}$-axis and $\bar{y}$-axis of MoCap. In Figure 4, the horizontal axis shows the $\bar{x}$ and $\bar{y}$ component of MoCap measurements in the left and right plot respectively and the vertical axis shows the accuracy after subtracting mean value from the measurements. Besides, the measurements are taken at different distances from the AprilTag in $\bar{y}$-axis, this information is coded in three colors such as red represents a distance of 30 cm, blue represents a distance of 50 cm and the green represents a distance of 70 cm. Plots in Figure 4 show that $\bar{x}$ component of MoCap measurements are more effected by viewing distance then $\bar{y}$ component. Further, as we move towards either the left or right side from the center of the tag, accuracy decreases.

**Figure 5:**
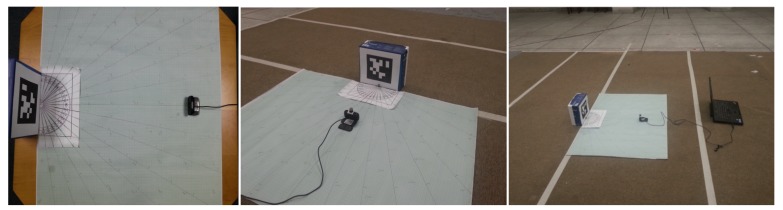
Photographs from different views of the AprilTag error measurement setup. (**Left**): Shows the top-down view of the error measurement setup. (**Middle**): Shows the placement of the camera in front of the AprilTag over error measurement setup. (**Right**): Shows the side view of the measurement recording process.

**Figure 6:**
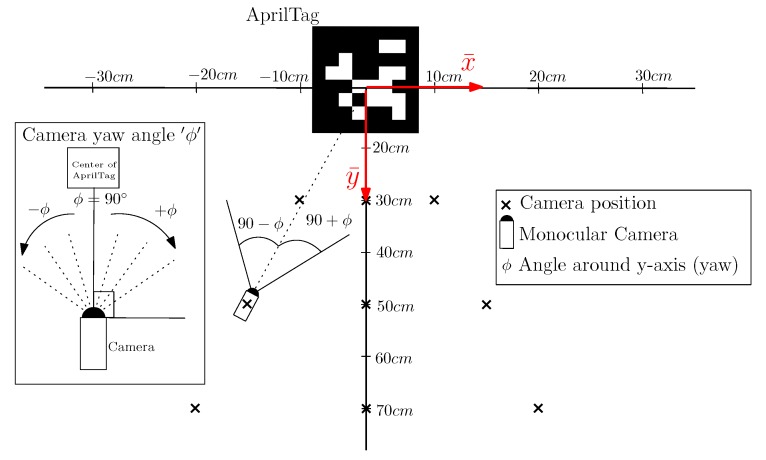
Error measurement setup showing measurement positions and yaw angles of the camera to AprilTag placed at the origin.

For the theoretical point of reference in experiments, we have used an error measurement markings to get a rough estimation regarding the position of the camera from the AprilTag, as shown in Figure 5. The design of the measurement experiments is illustrated in Figure 6, which shows how the readings have been taken to observe the actual inaccuracy caused by the various parameters such as distance and camera viewing angle. For the rest of the paper, analysis measurements are in a plane only, namely x-axis *x*, z-axis *z* and yaw angle ϕ in the camera frame of reference. The output of the measurements is represented in a 3-DOF AprilTag frame of reference τ with variables $\bar{x},\bar{y}$ and $\bar{\theta }$. In Figure 6, crosses represent the locations of the robot from which the readings are noted. This measurement technique is common for both the MoCap and the AprilTag data recordings for evaluation. These positions are obtained from the nominal reference points marked on an error measurement setup. The error measurement setup consists of a large paper sheet marked with angles and distances from the origin of AprilTag. At every nominal measurement point on error measurement setup, viewing yaw angle ϕ of the camera can be different. When the camera is pointing directly towards the center of the AprilTag, the yaw angle is $90^{\circ }$. If the camera is pointed towards the right side of the center, the yaw angle is 90+ϕ and for left, the yaw angle is 90−ϕ. We have taken measurements at 9 nominal points: three exactly in front of the tag and three on either side. The reason for selecting specific measurement points is to include maximum uncertainty in measurements for the viewing angles and the distances. Moreover, due to the limited field of view of the camera and the workspace environment, we keep the nominal points to 9 points. These points are uniformly covering each side and the face of the tag. Raw measurements at different angles and distances from AprilTag have been plotted and compared against the ground truth measured by MoCap.

**Figure 7:**
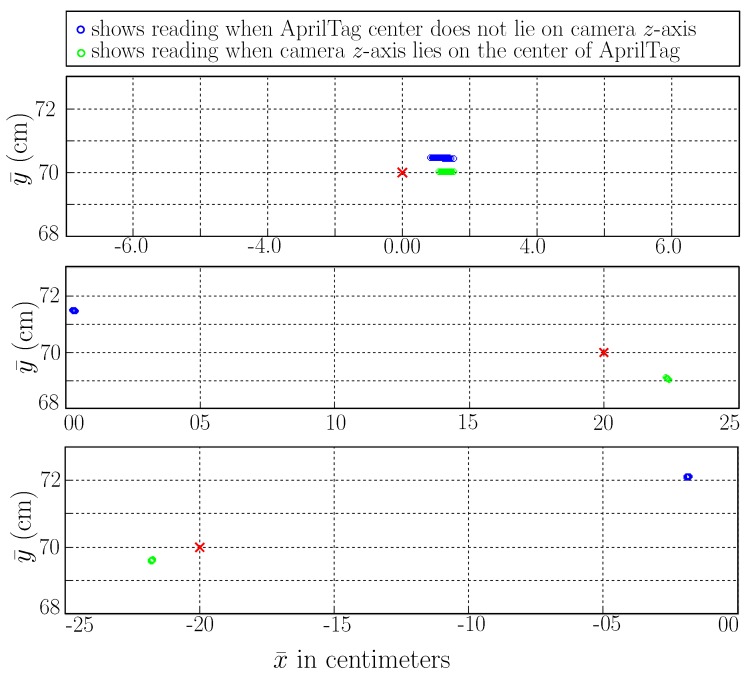
Multiple raw AprilTag readings plotted for ideal (green) and worst (blue) scenarios. Mean ground-truth (MoCap) readings are plotted as red crosses.

It is observed that the ideal scenario for AprilTag accuracy is when the camera is pointing towards the center of the tag or camera *z*-axis lies toward the center of AprilTag. Figure 7 shows the plots from raw measurements when the camera *z*-axis lies toward the center of AprilTag. The center of the tag is taken as $\left(\bar{x},\bar{y}\right)=\left(0,0\right)$ in AprilTag frame. These measurements are of the best accuracy that one can achieve from AprilTag and are used later as a reference. Similarly, Figure 7 also shows blue readings for which the camera *z*-axis does not lie towards the center of AprilTag. It can be seen that blue readings incur large inaccuracy when the camera is wider.

Table 3 summarizes the statistics of the measurements when the camera is pointing towards the center of the tag. For all readings, the yaw angle $\phi =90^{\circ }$ means it is directed towards the center of AprilTag. First two columns ‘$x_{r}$’ and ‘$y_{r}$’ show the *x*-axis and *y*-axis of nominal reference points where we wanted to place the camera. Third column ‘$\bar{x}$’ and forth columns ‘$\bar{y}$’ show ground truth values on the desired reference points using MoCap. Fifth column ‘*N*’ represents total number of readings taken at that reference point $\left(x_{r},y_{r}\right)$. Sixth column ‘$\mu_{\bar{x}}$’ and seventh column ‘$\mu_{\bar{y}}$’ give the mean in $\bar{x}$-axis readings and $\bar{y}$-axis. The eighth column ‘$\sigma_{\bar{x}}^{2}$’ and ninth columns ‘$\sigma_{\bar{y}}^{2}$’ show variances in $\bar{x}$-axis and $\bar{y}$-axis respectively. We get an average mean error of around 1.0 cm for $\bar{x}$ and around 0.40 cm error for $\bar{y}$ over a variable distance of ±6 cm, ±15 cm and ±70 cm in $\bar{x}$-axis and 30 cm, 50 cm and 70 cm in $\bar{y}$-axis.

**Figure 8:**
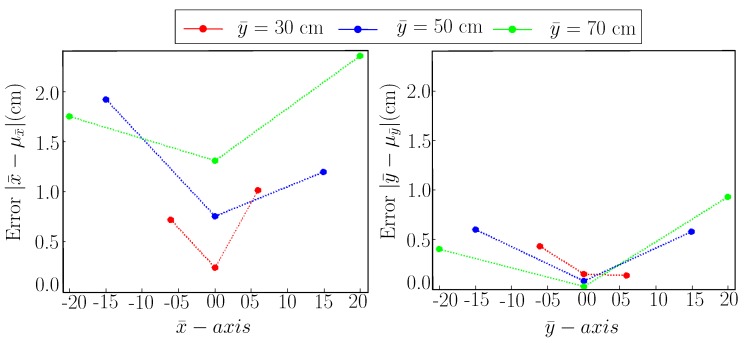
Error plot with camera’s *z*-axis pointed towards the center of AprilTag. (**Left**): Error plot for $\bar{x}$-axis. (**Right**): Error plot for $\bar{y}$-axis.

Figure 8 shows the mean error plot for the Table 3 using nominal reference points. We can see that the error is minimum for both $\bar{x}$ and $\bar{y}$ exactly in front of AprilTag. As we move along the left or right side, the error increases. Another notable finding is that the error increases as we increase the camera distance from the AprilTag along $\bar{y}$-axis.

**Figure 9:**
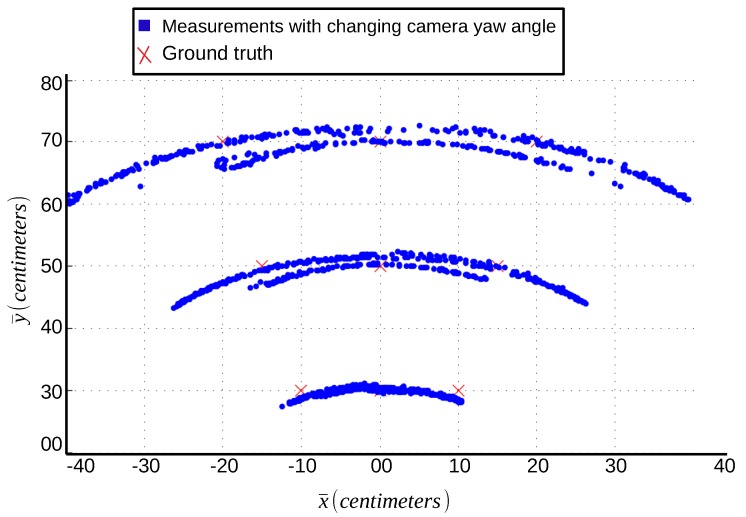
Plot for measurements with changing camera yaw angle ‘ϕ’ for $70^{\circ }\leq \phi \leq 110^{\circ }$.

Instead of pointing the camera towards the center of AprilTag, if we fix the camera such that its *z*-axis never points towards the center of the AprilTag, one gets the worst readings in terms of accuracy no matter which side of the tag the camera is located. To empirically analyze this concept, an experiment is performed in which measurements are taken at fixed measurement points with the varying camera yaw angle ϕ ranging from $70^{\circ }$ to $110^{\circ }$. Figure 9 shows the data plot of AprilTag with changing camera yaw angle ‘ϕ’. Here, the spread of data around a measurement reference point is in a circular path distributed almost evenly on both sides. Other than the plot representation, Table 4 shows the statistics of the reported data in terms of mean and variance in both measurement axis. As shown in Table 4, the variance $\left(\sigma_{\bar{x}}^{2},\sigma_{\bar{y}}^{2}\right)$ and mean $\left(\mu_{\bar{x}},\mu_{\bar{y}}\right)$ values of both $\bar{x}$ and $\bar{y}$ have increased manifold especially when $\bar{x}=\pm 20$ cm and $\bar{y}=70$ cm.

**Figure 10:**
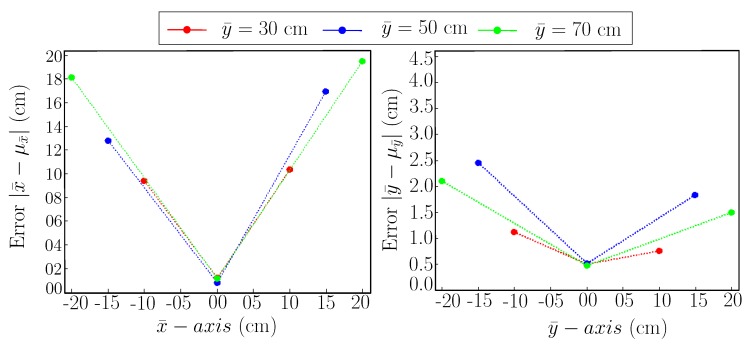
Error plot with camera yaw axis ‘ϕ’ fixed at $110^{\circ }$. (**Left**): Error plot for $\bar{x}$-axis. (**Right**): Error plot for $\bar{y}$-axis.

Additionally, Table 4 shows the mean value of all the measurements taken at a particular reference point with changing camera yaw angle ‘ϕ’. As Figure 9 shows, that the data spread is distributed almost evenly around the reference point along a circular path. In other words, it shows that for a particular reference point, as the camera yaw angle ‘ϕ’ changes, the reporting position also changes along the circular path of the distribution. Considering, the spread of data distribution is almost same on either side of the reference point, hence we get the mean values ($\mu_{\bar{x}},\mu_{\bar{y}}$) relatively near to the measurement reference point itself. To further analyze the worst possible case of camera yaw angle ‘ϕ’, a similar experiment has been conducted with camera yaw angle ‘ϕ’ fixed to $110^{\circ }$. Table 5 shows the statistical analysis of the experiment with the camera yaw axis fixed at ‘ϕ’=$110^{\circ }$. Table 5 shows that the inaccuracy has increased in the mean values ($\mu_{\bar{x}},\mu_{\bar{y}}$) especially in $\bar{x}$-axis. This is because the resulting measurements at ‘ϕ=$110^{\circ }$’ lie at the farthest sides of the circular spread shown in Figure 9. Moreover, Figure 10 shows the error plot for Table 5 against the ground truth(MoCap). It shows that the error is minimum at $\bar{x}=0$ but increases significantly as we move along the sides. For $\bar{y}=70$ cm, the error is around 16 cm for $\bar{x}=\pm 20$ cm whereas at $\bar{x}=0$ cm, the error is only around 2 cm.

Based on raw AprilTag measurements, the following shortcomings are identified in the current AprilTag implementation.

#### Distance from Tag:

It is observed that the accuracy decreases over distance as we move the camera away from the tag. As shown in Table 3 and Table 5, we can see the mean and variance for both $\bar{x}$ and $\bar{y}$ are increasing with increase in distance from the tag in z-axis.

#### Viewing Angle:

From multiple experiments, it is understood that the accuracy also decreases as the camera position changes from front to sideways. In the ideal scenario (Table 3), though the camera is pointing toward the center of the AprilTag at all the points, the error is less for $\bar{x}=0$ as compared to $\bar{x}\neq 0$. This error increases as we increase $\bar{x}$. Table 5 shows a similar pattern.

#### Yaw Angle of the Viewing Camera:

Previous extensive experiments show that the main source of inaccuracy is the frame inconsistency caused due to motion and significantly reduces the performance. The reason is that AprilTag fiducial system is coded in such a way that the output frame of reference is dependent upon the yaw angle ϕ orientation of the camera attached to the moving body. As the orientation of the moving body changes, the output frame also changes, making it hard to have a consistent frame of reference. At any given point, in *x* and *z* camera coordinates, change in yaw angle ϕ causes the generation of a new origin hence making a new frame of reference for every yaw angle. The new origin is the intersecting point of the AprilTag face plane with a straight line ‘*z*-axis’ from the center of the camera. So the current distance is reported under newly formed origin. Though the resulting output is relatively accurate in its respective frame of reference, the overall accuracy of all the yaw angles ϕ combined against a constant frame of reference is inaccurate. Figure 9 shows the plot of AprilTag reporting at fixed measurement points with varying yaw angle ϕ ranging from $70^{\circ }$ to $110^{\circ }$. Variance and mean readings of both $\bar{x}$ and $\bar{y}$ have also increased many folds as shown in Table 4 especially when $\bar{x}=\pm 20$ cm and $\bar{y}=70$ cm.

## 4. Improvement Techniques

Based on the measurement analysis of the AprilTag system, the following improvement techniques have been proposed.

### 4.1. Passive Correction for Frame Consistency

**Figure 11:**
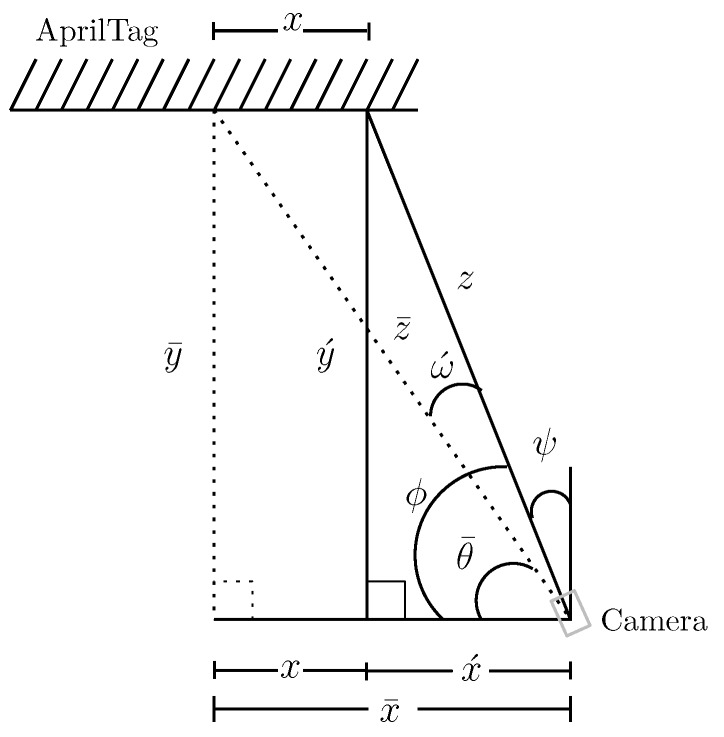
Geometrically aligning subsequent frames.

As illustrated in Section 3.3, the key source of inaccuracy in AprilTag readings is the misalignment of the camera *z*-axis with the center of the tag. When the camera follows a certain trajectory, its orientation may change over time, which causes inconsistency between two consecutive frames. To solve this problem, we propose a passive-orientation correction. Also referred to as a “Soft Yaw Axis Correction (SYAC)” technique. In this technique, the geometry of the whole setup is modified in a way that the axis (*z*-axis) passing through the center of the camera always points towards the tag’s origin that is, $\left(\bar{x},\bar{y}\right)=\left(0,0\right)$. Figure 11 shows the drawing for trigonometric correction. The solid triangle shows the original geometry without any correction in the camera frame of reference. The hypotenuse of a solid triangle *z*, which emerges from the camera center, should touch the center of tag. That ideal line is called $\bar{z}$, depicted as a dotted line in Figure 11. Moreover, ϕ is known, the angle $\overset{´}{\omega }$ that aligns the dotted triangle hypotenuse with the center is calculated. By using simple trigonometry, $\bar{z}$ and $\overset{´}{\omega }$ is calculated as:

(3) $$\overset{´}{\omega }=\phi -\tan^{-1}\left(\frac{z\sin\phi }{x+z\cos\phi }\right),$$

(4) $$\bar{z}=\sqrt{{\left(\left(z\sin\phi \right)\right)}^{2}+{\left(\left(x+z\cos\phi \right)\right)}^{2}}.$$

Once $\bar{z}$ and $\overset{´}{\omega }$ are known, $\bar{x}$ Equation (5), $\bar{y}$ Equation (6) and $\bar{\theta }$ Equation (7) are derived which eventually improves the accuracy.

(5) $$\bar{x}=x+\overset{´}{x}=x+z\cos\phi ,$$

(6) $$\bar{y}=z\sin\phi ,$$

(7) $$\bar{\theta }=\arctan\left(\frac{\overset{´}{y}}{x+\overset{´}{x}}\right)=\arctan\left(\frac{z\sin\phi }{x+z\cos\phi }\right).$$

**Figure 12:**
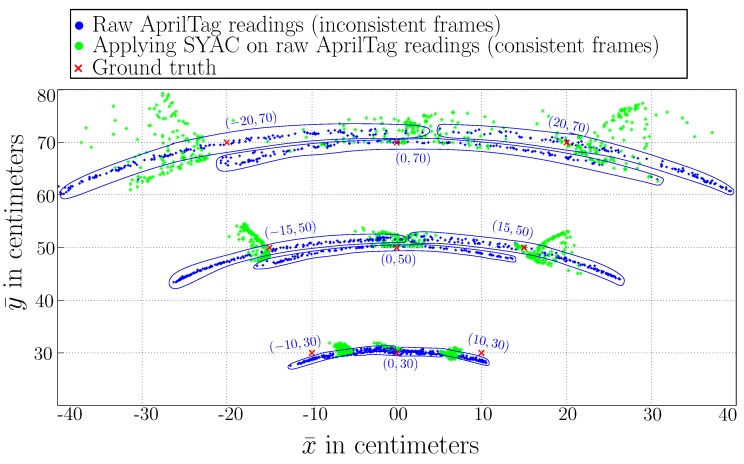
A comparison plot for AprilTag raw readings and improved SYAC measurements with changing camera yaw angle ‘ϕ’ for $70^{\circ }\leq \phi \leq 110^{\circ }$. Blue circles show the clustering of the plotted data around a ground truth point.

Figure 12 shows the data scatter plot after applying this passive correction technique. It can be seen that the spread of the transformed data is decreased and Table 6 shows decreased variance both in $\bar{x}$ and $\bar{y}$ axis. By zooming point (x,z)=(0.20,0.70), it can be observed that the original readings are displaced only after applying the correction, making them more closely to the reference point. At camera yaw angle of $110^{\circ }$, it is almost 40 cm off the true position in $\bar{x}$-axis and 2 cm in $\bar{y}$-axis. After applying the correction, the error in $\bar{x}$-axis is reduced to 5 cm and in $\bar{y}$-axis to 1 cm. Similarly, at yaw angle, $70^{\circ }$ in $\bar{x}$-axis, the error is reduced from 26 cm to 4 cm.

**Figure 13:**
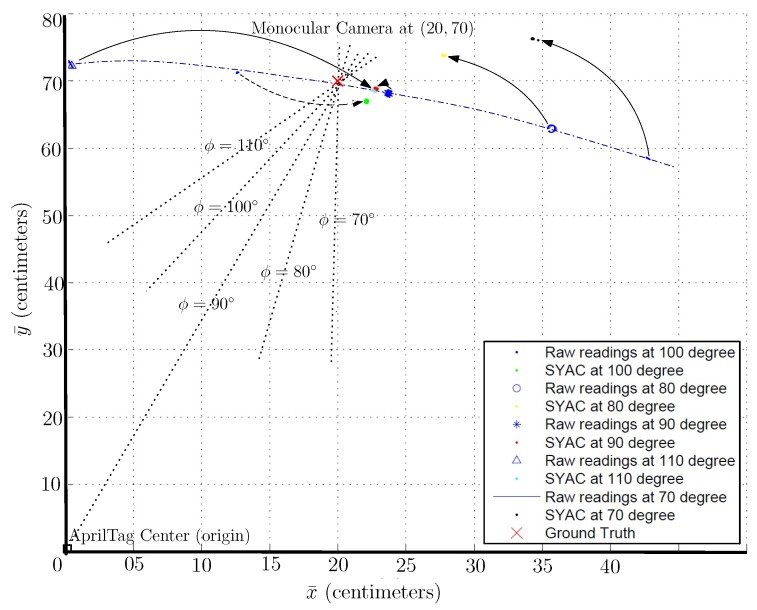
An angle-wise comparison plot for AprilTag raw readings and improved SYAC measurements with changing camera yaw angle ‘ϕ’ for $70^{\circ }\leq \phi \leq 110^{\circ }$. Plot shows that our proposed technique has significantly improved AprilTag raw measurements.

To further extend the comparison, Figure 13 shows the improvement of AprilTag readings concerning the camera yaw angle ’ϕ.’ The rotation of the camera around its yaw axis is limited to five sampling angles that is, $70^{\circ }$, $80^{\circ }$, $90^{\circ }$, $100^{\circ }$ and $110^{\circ }$. The rate of rotation for yaw angle ’ϕ’ is 10 degrees/sec. Hence, it takes the camera 5 seconds to sweep in one direction. Moreover, AprilTag is being detected at 11 Hz; hence, we have approximately 11 readings at an individual yaw angle ϕ during a single sweep. As Figure 13 shows that at each measurement angle ’ϕ,’ our proposed Soft Yaw Axis Correction approach (SYAC) has significantly improved the accuracy of raw AprilTag. Red cross $\left(\bar{x},\bar{y}\right)=\left(20,70\right)$ shows the ground-truth value for the whole experiment. As we can see from the plot that the accuracy of AprilTag decreases as we increase the yaw axis angle ’ϕ’ of the camera. The accuracy is worse when ’ϕ’ is either $110^{\circ }$ or $70^{\circ }$. As the camera yaw angle ’ϕ’ approaches $90^{\circ }$, which implies the camera’s *z*-axis points towards the center of the tag, accuracy increases. As a result, Figure 13 shows data at ’$\phi =90^{\circ }$’ most accurate.

### 4.2. Active Correction with a Yaw Axis Gimbal

**Figure 14:**
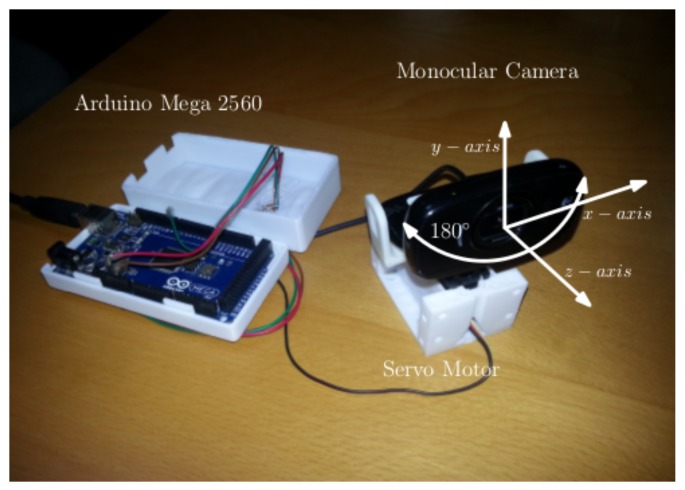
Yaw-axis gimbal hardware setup developed by the authors. A monocular camera has been mounted on a Dynamixal stepper motor, which is controlled by an Arduino Mega 2560 controller. The controller is used as a slave ROS process in localization application. Housing is in a 3D printed retrofit.

**Figure 15:**
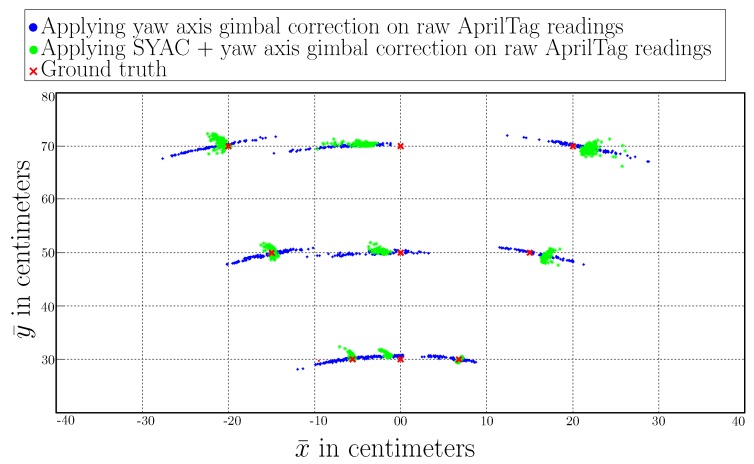
Data scatter plot for geometrically consistent (SYAC) and non consistent frames(raw AprilTag) with custom-built yaw axis gimbal.

Another way to correct for misalignment of camera *z*-axis with the center of the tag is to track and correct it in real-time using a yaw axis gimbal actively. The custom-built hardware setup is proposed to achieve this, as shown in Figure 14. The tracking Algorithm 1 consists of a Proportional-Integral-Derivative (PID) based action tracking controller working at 10Hz. The input to the Algorithm 1 is a raw yaw angle of the camera ‘ϕ’ in the camera frame of reference reported by native AprilTag implementation. As discussed in Section 3.3, the yaw angle of the camera ‘ϕ’ depends upon the alignment of the camera *z*-axis with the center of AprilTag. If the camera *z*-axis lies on the center of the AprilTag, ‘ϕ’ is equal to zero. As the face of the camera moves away from the center of the AprilTag, ‘ϕ’ value changes and introduces inaccuracy. Moreover, the goal of the Active Correction with a Yaw Axis Gimbal (ACYG) is to keep the *z*-axis of the camera aligned towards the center of the tag by keeping angle ‘ϕ’ equals to zero. Significant improvement in the tag’s precision has been observed with almost similar accuracy by using this technique. One of the problems with the Passive Correction for Frame Consistency technique is that it does not align the camera center accurately towards the center of the tag if the yaw angle ϕ is too big. Therefore, active compensation in combination with a passive correction ensures that the camera yaw angle does not become too big. Figure 15 shows the effect of one-axis tracking gimbal in the combination of passive frame consistent Correction and without passive frame consistent Correction. Data scatter plots show the accuracy has increased significantly, especially in combination with SYAC. Table 7 and Table 8 summarize the variances and mean values while using yaw axis gimbal with raw AprilTag and with SYAC correction, respectively.

Figure 15 shows that though active correction has improved the overall accuracy. If this technique is applied with a combination of passive correction (SYAC), the resultant readings are more accurate. The reason behind this is that both the correction methods have their limitations. In Soft correction (SYAC), sometimes the camera *z*-axis fails to align with the tag center if the measured yaw angle ’ϕ’ of the viewing camera is too large. Moreover, in Active correction, the tag is being detected at 11 Hz and the active correction is being done at 8 Hz to due system limitations. Hence, this results in the incursion of inaccuracy in the active correction system. Henceforward, by using both correction techniques in combination with each other improves the overall accuracy manifolds.
**Algorithm 1** Active Camera Tracking of AprilTag Center.**Input:** camera yaw angle ’ϕ’ **Output:** servo angle ’Γ’
1:$K_{P}\leftarrow$ Propotional gain depending upon *z*-axis value.2:$K_{I}$ Integral gain depending upon *z*-axis value.3:$K_{D}\leftarrow$ Propotional gain depending upon *z*-axis value.4:ϵ← Initialize error with zero5:T← 0.05 ▹ Servo stopping threshold.6:α← 0.008 ▹ Smoothing factor. 7:ϵ←ϕ8:**if**ϵ>T**then**9:Integral ← Integral + ϵ10:**else** Integral ← 0.0011:**end if**12:P ←ϵ$\times K_{P}$13:I ← Integral $\times K_{I}$14:D ← (LastYawAngle − CurrentYawAngle) $\times K_{D}$15:Drive ← P + I + D16:Drive ← Drive ×α17:**if** Drive > 90 **then**▹ To keep camera facing AprilTag18:    Drive ← 9019:**else** Drive < -9020:    Drive ← -9021:**end if**22:Γ← CurrentServoAngle + Drive23:LastYawAngle ← CurrentYawAngle24:CurrentServoAngle ←Γ25:**return**Γ

### 4.3. Comparative Results

Following the extensive experimentation and dataset collection, comparative results have been deducted to show the improvement more comprehensively. Table 9 shows the error comparison of different approaches against AprilTag. Here error is represented as the difference between the reported mean value and the ground truth both in $\bar{x}$ and $\bar{y}$ axis. Columns 1 and 2 show the ground truth (MoCap) values of $\bar{x}$ and $\bar{y}$, respectively, against which the standard error is compared. Columns 3 and 4, which are labeled as “Raw AprilTag readings (camera pointing towards tag’s center),” show the error for the AprilTag system when the camera is always pointed towards the center of tag. Earlier experiments have shown that this is the maximum possible accuracy one can achieve using AprilTag. Moreover, columns 5 and 6 “Raw AprilTag readings (camera pointing away from tag’s center)” shows the raw data from AprilTag when the camera *z*-axis is not aligned with the center of the tag resulting in inconsistent frames induced by camera motion. Columns 7 and 8, which are labeled as “Applying Soft Yaw Angle Correction (SYAC) on raw AprilTag readings,” shows mean error after applying the proposed approach of Soft Yaw Angle Correction (SYAC) to make the inconsistent frames consistent. Similarly, columns 9 and 10 labeled as “Applying Active Correction with Yaw Axis Gimbal on raw AprilTag readings” show error after using custom build yaw axis gimbal on raw AprilTag system. Lastly, columns 11 and 12, which are labeled as “Applying (SYAC + Active Yaw Axis Gimbal correction) on raw AprilTag readings,” show an error when both proposed approaches of soft and active yaw axis correction are applied in combination.

**Figure 16:**
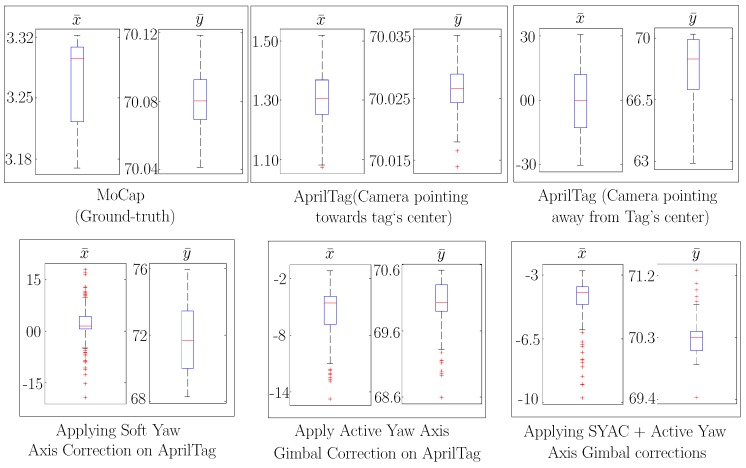
Comparison of resulting data spread (precision) from different approaches against the ground truth (Mocap) at nominal reference point straight in front of AprilTag i.e., $\left(\bar{x},\bar{y}\right)=\left(0,70\right)$.

**Figure 17:**
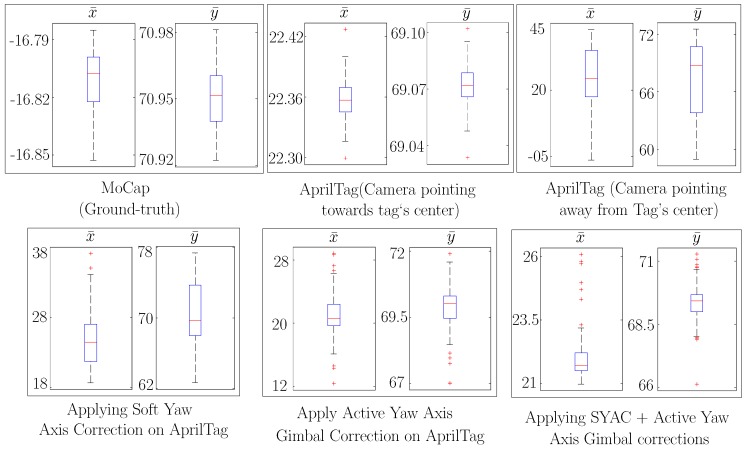
With an oblique viewing angle i.e., $\left(\bar{x},\bar{y}\right)=\left(20,70\right)$, a comparison of resulting data spread (precision) from different approaches against the ground truth (Mocap).

Additionally along with the accuracy, precision of the AprilTag system has also been increased manifolds by our proposed approaches as shown in Figure 16 and Figure 17. Figure 16 shows the resulting precision of different approaches in cm for a nominal reference point of $\left(\bar{x},\bar{y}\right)=\left(0,70\right)$. We can see that the spread of $\bar{x}$ for raw AprilTag data with camera’s *z*-axis not pointing towards tag’s center is around 13.9 cm while after applying Soft Yaw Angle Correction (SYAC) plus Active Yaw Axis Gimbal Correction, it is decreased to 1.27 cm. Moreover for $\bar{y}$, the spread is decreased from 1.61 cm to 0.22 cm. In addition, Figure 17 shows the similar analysis for nominal reference point of $\left(\bar{x},\bar{y}\right)=\left(20,70\right)$. Here after applying Soft Yaw Axis Correction (SYAC) and Active Yaw Axis Gimbal Correction on AprilTag, the precision has improved manifolds and the data spread for $\bar{x}$ and $\bar{y}$ is decrease from 12.04 cm to 0.84 cm and 3.74 cm to 0.65 cm respectively. Nonetheless, Motion Capture (MoCap) spread has also been illustrated for both the nominal reference points for ground-truth analysis.

**Figure 18:**
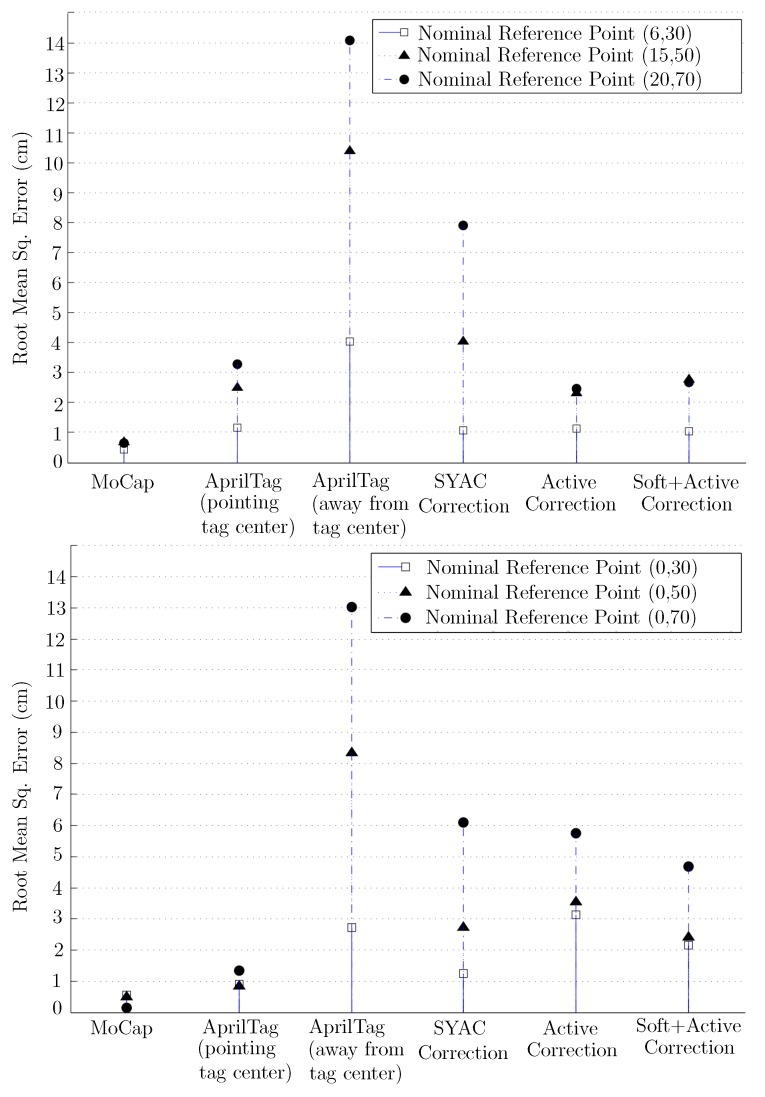
Root Mean Square Error (RMSE) comparison of raw AprilTag against proposed approaches and MoCap.

As mentioned earlier, the objective is to reduce the measurement error close to the ground truth. Figure 18 shows the statistical analysis of accuracy by plotting Mean Root Square Error (RMSE) achieved by the proposed approaches against the raw AprilTag. It shows that our proposed approaches have significantly reduced the RMSE as compared to bare AprilTag results. Moreover, this error is further reduced when both the proposed approaches are combined. The resulting error is significantly close to ground-truth and the ideal scenario when the camera is pointing towards the center of AprilTag hence achieving our objective.

Furthermore, results from Table 9, Figure 16 and Figure 17 have shown that we can achieve significant improvements in the accuracy and precision of the AprilTag by the slight trade-off with execution time. Table 10 shows an average execution time for a single input frame for different approaches. Nevertheless, the raw implementation of AprilTag has the quickest execution time as compared to the proposed approaches but the difference is not significant. As illustrated by Table 10, a combination of both proposed methods (Passive yaw axis correction + Active correction with yaw axis gimbal) can achieve a maximum operating frequency of approx. 4 Hz, which is acceptable for most of the robotics applications.

### 4.4. Probabilistic Sensor Model for AprilTag

The third contribution of this paper is the development of a forward probabilistic sensor model p(Y|X) for the AprilTag. The model is based on the collected measurement data and works for all the locations. These locations include both direct and indirect measurement points. Hence, it makes the empirical analysis of the current work applicable to a probabilistic decision-theoretic framework. With reference to Figure 2 and Figure 11, the true state *X* of the robot is given by the tuple $X={\left[\bar{x},\bar{y},\bar{\theta }\right]}^{T}$. The measurement vector *Y* is also a triplet $Y={\left[z,x,\phi \right]}^{T}$. The measurements are assumed to be a nonlinear transformation of the true state, corrupted by some additive sensor noise, Y=F(X)+ε. Explicitly, these relations can be written as:

(8) $$z=\sqrt{{\left(\bar{z}\cos\bar{\theta }-x\right)}^{2}+{\left(z\sin\phi \right)}^{2}}+\varepsilon_{z},$$

(9) $$x=\bar{z}\cos\bar{\theta }-z\cos\phi +\varepsilon_{x},$$

(10) $$\phi =\arctan\left(\frac{\bar{z}\sin\bar{\theta }}{\bar{z}\cos\bar{\theta }-x}\right)+\varepsilon_{\phi }.$$

We are interested in finding the joint probability distribution of the measurement vector given the true states $p\left(z,x,\phi |\bar{x},\bar{y},\bar{\theta }\right)$. In order to find the above mentioned probability, Bayes’ theorem is applied to obtain:

(11) $$p\left(\bar{x},\bar{y},\bar{\theta }|z,x,\phi \right)=\frac{1}{J}\;p\left(z,x,\phi |\bar{x},\bar{y},\bar{\theta }\right)p\left(x,z,\phi \right),$$

where *J* is a constant that can be factored out. Since we have no prior distribution p(x,z,ϕ), one can use a Maximum Likelihood Estimator for a uniform prior, that is, a simple inversion of the model to deduce the states from the measurements by using Equations (5)–(7).

Hence, if we have a model $p\left(\bar{x},\bar{y},\bar{\theta }|z,x,\phi \right)$ for *all* states, we can use it to localize at even points where we do not have measurement data. We achieve this using a Gaussian Process (GP) based regression method [44] as follows. First we make a simplifying assumption that *Y* are not mutually correlated. While this may not be factually true, we find below that this is sufficient for using the Tag in practice. (The extension of the framework to correlated sensor measurements is a work in progress.) Therefore, we focus on either of the measurement variable in *Y* as scalar nonlinear transformations f(X). These are precisely the individual measurement equations given above. Using the notation introduced in Reference [44], we are interested in finding the distribution $p(f_{*}^{test}|X,X_{*},Y_{*})$, where $f_{*}^{test}$ is a stochastic process for which $\bar{x},\bar{y}$ and $\bar{\theta }$ has a joint Gaussian distribution, $X=(\bar{x},\bar{y},\bar{\theta })$) is the unknown test point where the distribution has to be calculated, $X_{*}$ are the ground truth points for training data $X_{*}\in {\left\{\left({\bar{x}}_{i},{\bar{y}}_{i},{\bar{\theta }}_{i}\right)\right\}}_{i=1}^{N}$ and $Y_{*}\in {\left\{\left({\bar{x}}_{i},{\bar{y}}_{i},{\bar{\theta }}_{i}\right)\right\}}_{j=1}^{M}$ are the data collected in experiments as output of AprilTag at training points $X_{*}$.

In GP regression, we have to define a covariance function (or Kernel function) whose parameters (the so-called hyper-parameters) have to be tuned to best explain the data at hand. We have chosen a squared exponential covariance function, which is widely used because of its smoothness and differentiability:

(12) $$k\left(x_{a},x_{b}\right)=\alpha \exp^{\frac{-|x_{a}-x_{b}{|}^{2}}{2\beta }}$$

where α and β are the hyper-parameters of the kernel function.

The GP regression methodology makes the assumption that the training output $Y_{*}$ and test output $f_{*}^{test}$ have a joint Gaussian distribution.(Once again, this is a simplifying assumption that may be invalid in practice but works in practice.)

(13) $$\left[\begin{array}{c} Y_{*}\\ f_{*}^{test} \end{array}\right]∽N\left(0,\left[\begin{array}{cc} K(X_{*},X_{*}) & K(X_{*},X)\\ K(X,X_{*}) & K(X,X) \end{array}\right]\right)$$

where $K(X_{*},X_{*})$ is a N × N matrix defined by covariance function (kernel) evaluated at every training point $X_{*}$ against each training point $X_{*}$. K(X,X) is an M×M matrix defined by kernel evaluated at every test point *X* against each test point *X*. $K(X_{*},X)$ is an N×M matrix formed by the kernel evaluated at every training point $X_{*}$ against each test point *X*. Further details can be seen in a standard references on GP (e.g., Reference [44]).

Here, we are only interested in incorporating the knowledge provided by the training data $X_{*}$ regarding distribution functions other than drawing random functions from prior knowledge. So we will restrict the joint prior distribution to contain only those functions which agrees with the observed data points $Y_{*}$ to get the posterior distribution over functions. In other words, we will reject all those functions generated from prior that disagrees with the observations. In probabilistic terms, this can be achieved by marginalizing the observations over the joint distribution to get the predicted distribution as $p\left(f_{*}^{test}|X,X_{*},Y_{*}\right)∽N\left(\mu ,\Sigma \right)$, where

(14) $$\begin{array}{c} \mu =K\left(X,X_{*}\right){\left(K\left(X_{*},X_{*}\right)+\sigma_{A}^{2}I\right)}^{-1}Y_{*},\\ \;\Sigma =K\left(X,X\right)-K\left(X,X_{*}\right){\left(K\left(X_{*},X_{*}\right)+\sigma_{A}^{2}I\right)}^{-1}K\left(X_{*},X\right)), \end{array}$$

where $\sigma_{A}^{2}$ is the noise variance for the particular AprilTag measurement variable under consideration. The process is repeated for all three measurement variables to regress the distribution for all measurement-state pairs. The results of the regression have been summarized in Table 11.

#### Experimental Verification of Sensor Model

To verify the validity of our proposed AprilTag sensor model, we have used our sensor model in various settings to estimate the state of a robot. We assume a standard odometry model in which robot can rotate around its axis and can move forward [45]. We have performed both indoor and outdoor experiments to validate our proposed sensor model.

For indoor experiment, at any time step *t* state vector $X_{t}$ is given by $X_{t}={\left[\bar{x},\bar{y},\bar{\theta }\right]}^{T}$, where $\bar{x}$ shows the movement of robot in x-axis, $\bar{y}$ show is the movement in y-axis and $\bar{\theta }$ shows the rotation around robot‘s own axis. Our goal is to find $p(X_{t}|X_{t-1},u_{t},z_{t})$ where $X_{t-1}$ is the robot state in previous time stamp, $u_{t}$ is the current input command and $z_{t}$ is the current sensor measurement.

We have used Monte Carlo simulation technique [46] to estimate the position and pose of the robot since it does not require any prior knowledge for data distribution. In this method, *k* number of particles are randomly generated around an initial starting point $X_{i}$ with certain initial uncertainty based upon system

(15) $$X_{p}∼N\left(\left[\begin{array}{c} x_{i}\\ y_{i}\\ \theta_{i} \end{array}\right],\left[\begin{array}{ccc} \sigma_{xx}^{2} & 0 & 0\\ 0 & \sigma_{yy}^{2} & 0\\ 0 & 0 & \sigma_{\theta \theta }^{2} \end{array}\right]\right),$$

where $X_{p}$ are the randomly generated particles and p∈{1,...,k}, $x_{i}$ is the initial value for x-axis, $y_{i}$ is the initial value for y-axis, $\theta_{i}$ us the initial angle and $\sigma_{x}^{2},\sigma_{y}^{2},\sigma_{\theta }^{2}$ are the initial variance in *x*-axis, *y*-axis and θ respectively. Then, each particle is propagated forward based upon the motion model assumed

(16) $$X_{t}=f_{t}\left(X_{t-1}\right)+n=f_{t}\left(x_{t-1},y_{t-1},\theta_{t-1}\right)+n,$$

where $f_{t}$ is a function representing motion model of the system and *n* is the Gaussian noise. Then observation model is applied on each propagated particle to get observation measurements as ${\hat{z}}_{t}$. Then these observation measurements are weighted against the measurement data from the sensor $z_{t}$. Each particle is assigned a probabilistic weight based upon how close it is to the measurement after applying observation model as

(17) $$P_{weight}^{p}=\frac{1}{\sqrt{{\left(2\pi \right)}^{3}\det R}}*exp\left(-\frac{1}{2}\left(z_{t}-{\hat{z}}_{t}\right)R^{-1}{\left(z_{t}-{\hat{z}}_{t}\right)}^{T}\right),R=\left[\begin{array}{ccc} r_{x} & 0 & 0\\ 0 & r_{y} & 0\\ 0 & 0 & r_{\theta }, \end{array}\right]$$

where p∈{1,...,k} represents number of particles, *R* is a 3 × 3 co-variance matrix. Then the assigned probability weights are normalized such that their sum is equal to 1 as

(18) $$P_{CDF}=\frac{P_{weight}^{p}}{\sum_{n=0}^{k}P_{weight}^{n}},$$

where $P_{CDF}^{p}$ is the cumulative distribution of the probability density of weighted vector $P_{weight}^{p}$. Then weighted particles are re-sampled for the next step by uniformally sampling from the cumulative distribution as shown in Equation (19). Since the particles are being selected by statistical probabilities, so on average, particles with the greater weights are being selected.

(19) $$X_{p}=P_{CDF}^{-1}\left(h\right),\;\mathrm{where}\;h∼U\left(0,1\right).$$

After getting new particles, the whole process is repeated for $m_{o}$ times where $m_{o}$ is the total number of tag observations observed in an experiment. At every step, the average of all the particles is considered to be the true position of the robot. This algorithm relies on the survival of the fittest philosophy. Those particle which are close to the sensor measurement are weighted higher then others give them the chance to be selected again for the next round.

**Figure 19:**
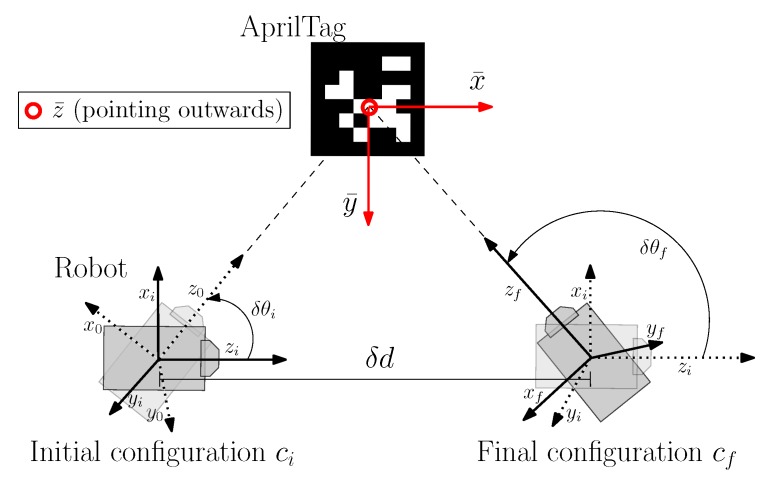
Incremental motion model used between two configuration points $c_{i}$ and $c_{f}$, encoded by three parameters $\delta \theta_{i}$, δd and $\delta \theta_{f}$ for Monte Carlo simulation.

In our experiment, an incremental motion model has been used for the propagation of particles from one configuration $c_{i}$ to another configuration $c_{f}$. Three parameters $\delta \theta_{i}$, δd and $\delta \theta_{f}$ have been used to encode the complete motion from one configuration to another. Input command $u_{\theta_{i}}$ maps as rotation $\delta \theta_{i}$ of robot at initial configuration $c_{i}$ such that it faces final configuration $c_{f}$. $u_{d}$ maps as the straight forward motion δd from initial configuration $c_{i}$ to final configuration $c_{f}$ and $u_{\theta_{f}}$ maps the final rotation $\delta \theta_{f}$ at destination point for final pose angle. See Figure 19 which shows each parameter in detail.

For proposed sensor model verification using Monte Carlo simulation, we used 10,000 particles initially generated at a known starting point $X_{i}=\left[x_{i},y_{i},\theta_{i}\right]$ with initial variance of $n_{i}$. As it is assumed earlier, our robot can only move forward and can rotate around its own axis, so motion model for each particle can be given by

(20) $$\begin{array}{c} X_{t}=\left[\begin{array}{c} x_{t-1}+d_{for}\cos\left(\theta_{t-1}+\delta \theta_{i}\right)+n_{x}\\ y_{t-1}+d_{for}\sin\left(\theta_{t-1}+\delta \theta_{i}\right)+n_{y}\\ \theta_{t-1}+\delta \theta_{i}+\delta \theta_{f}+n_{\theta } \end{array}\right], \end{array}$$

where $d_{for}$ is the forward distance as a result input command $u_{for}$, $\delta \theta_{i}$ is the angle of rotation at initial position as a result of input command $u_{\theta_{i}}$, $\delta \theta_{f}$ is the rotation angle at final configuration point as a result of input command $u_{\theta_{f}}$. $n_{x}$ is the Gaussian noise in x-axis, $n_{y}$ is the Gaussian noise in y-axis and $n_{\theta }$ is the Gaussian noise in θ.

At any time *t*, measurement vector is given by $z_{t}={\left[x_{tag},y_{tag},\theta_{tag}\right]}^{T}$. In this experiment, we have assumed that *x*, *y* and θ are independent in nature. So for observation model, the proposed AprilTag sensor model as shown in Equation (14) has been used.

**Figure 20:**
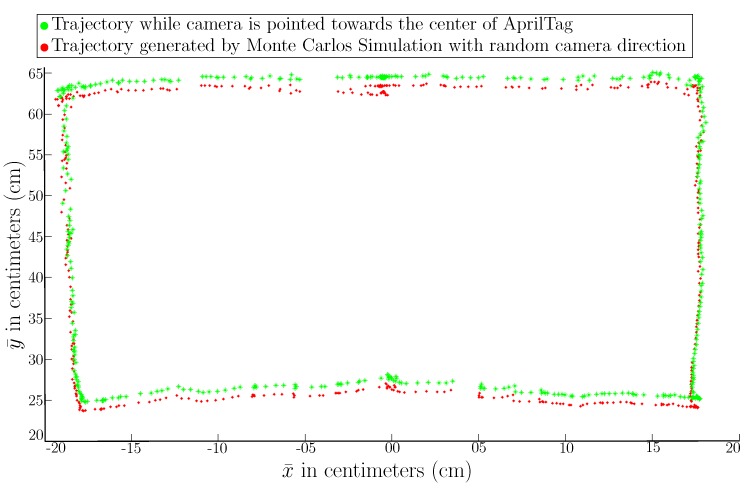
Trajectory comparison between MoCap and trajectory generated by Monte Carlo simulation using our proposed AprilTag sensor model.

Figure 20 shows the trajectory generated by applying the particle filter empowered with our proposed AprilTag sensor model in comparison with the ground truth generated by MoCap. AprilTag‘s center is placed at $\left(\bar{x},\bar{y},\bar{\theta }\right)=\left(0,0,90\right)$ over a calibrated setup and robot is moved in front of AprilTag in a rectangular shape. The rectangular shape is selected to have a better visualization of trajectory data and to see the loop closure. Figure 20 shows that the trajectory generated by the particle filter (red) is very close to the ground truth trajectory (green). The experiment shows that the particles converge very quickly because of the high precision of the system achieved by applying proposed techniques.

**Figure 21:**
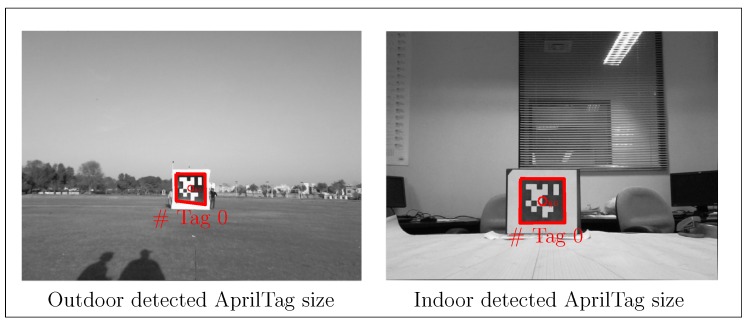
(**Left**): Camera view of detected AprilTag (red polygon) in outdoors. (**Right**): Camera view of detected AprilTag (red polygon) in indoors. Both images show detection polygons along with the detected tag IDs based on implanted code.

**Figure 22:**
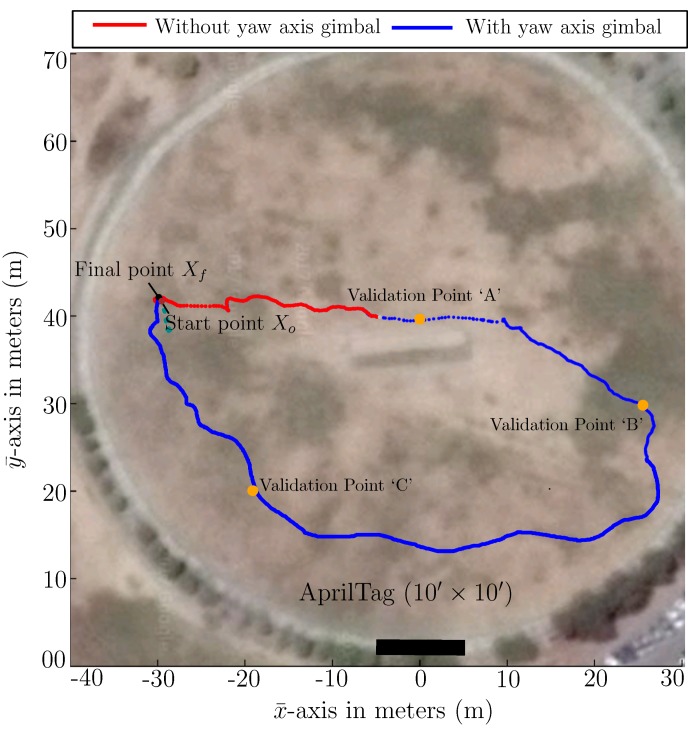
Trajectory generated using Monte Carlo Simulation in an outdoor environment.

To further investigate the performance of our proposed sensor model, a similar experiment in a large outdoor environment has been performed. For this purpose, a larger AprilTag of size 305×305 cm fixed on the ground has been used, as shown in Figure 21. In this experiment, the robot has moved along an irregular path from the left side of the AprilTag to the right side as far as the tag is visually detectable and then back to the left side towards starting position, as shown in Figure 22. To show the significance of each proposed improvement, we have divided the experiment into two phases. In phase one, active tracking of AprilTag is not activated and only passive correction is done using the sensor model (red path). In phase two, active tag tracking is also activated along with the passive correction (blue path), as shown in Figure 22. Considering the experiment is in an outdoor environment, therefore ground truth trajectory can not be generated. Hence for ground truth verification, we have manually marked three validation points in meters that is, $A\left(\bar{x},\bar{y}\right)=\left(0,40\right)$, $B\left(\bar{x},\bar{y}\right)=\left(26,30\right)$ and $C\left(\bar{x},\bar{y}\right)=\left(-19,20\right)$ before the experiment and have deliberately passed through them. Figure 22 shows that the trajectory passes through the validation points.

Moreover previously proposed pose-indexed probabilistic sensor model in Equation (14) is regressed over an indoor small scale experimental data. The training points are at the maximum of 1 m from the AprilTag. Therefore, the model trained by using Gaussian Processes(GP) 14 is only valid for sub-meter trajectories. To make it workable in long distances, we have proposed a general sensor model with some scale factor *d*, where *d* is the distance of the camera from the tag along the $\bar{y}$-axis. To calculate the scale factor *d*, we have used the equality as shown in Equation (21).

(21) $$d=\frac{f\times h_{r}\times I_{p}}{h_{p}\times S_{s}\times c}$$

where *f* is focal length of camera in mm, $h_{r}$ is the real height of the AprilTag in mm, $I_{p}$ is the height of image sensor in pixels, $h_{p}$ is the AprilTag height in pixels and $S_{s}$ is the image sensor‘s height in mm. *c* is a constant to change the unit scale. Since for outdoor experiment, wehave used meters as our unit of choice for distances, so we have used c=1000. After evaluating scale factor *d*, our general sensor model would become:

(22) $$\begin{array}{c} \mu_{G}=K_{G}\left(X,X_{*}^{G}\right){\left(K_{G}\left(X_{*}^{G},X_{*}^{G}\right)+\sigma_{A}^{2}I\right)}^{-1}Y_{*}^{G},\\ \Sigma_{G}=K_{G}\left(X,X\right)-K\left(X,X_{*}^{G}\right){\left(K_{G}\left(X_{*}^{G},X_{*}^{G}\right)+\sigma_{GA}^{2}I\right)}^{-1}K_{G}\left(X_{*}^{G},X\right)),\\ K_{G}=k_{G}\left(x_{a},x_{b}\right)=d^{2}\times \alpha \exp\left(\frac{-|x_{a}-x_{b}{|}^{2}}{2\times d^{2}\times \beta }\right),\;\;\\ X_{*}^{G}=X_{*}\times d,\;Y_{*}^{G}=Y_{*}\times d\;,\;\sigma_{GA}^{2}=\sigma_{A}^{2}\times d^{2}. \end{array}$$

Here $\mu_{G}$ and $\sigma_{G}$ is the mean value and variance for test point *X* using generalized sensor model respectively. $K_{G}$ is the generalized kernel, $X_{*}^{G}$ is the generalized training point and $Y_{*}^{G}$ is the generalized observed value. We have empirically tested and verified experimentally that the generalized sensor model gives almost same result at certain distance ’*d*’.

**Figure 23:**
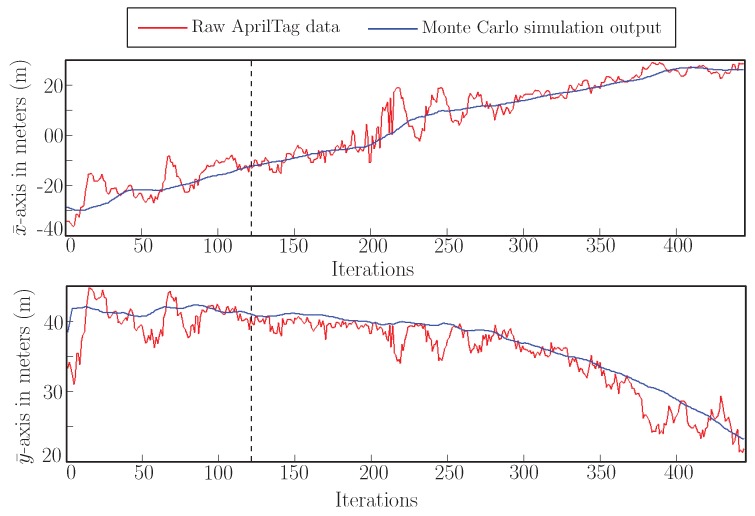
Comparison of raw AprilTag data and proposed generalize sensor model based particle filter output along $\bar{x}$-axis and $\bar{y}$-axis. Dotted line shows the initialization of yaw axis gimbal for active correction.

Figure 22 shows the trajectory generated by our generalized sensor model in an outdoor environment by using Monte Carlo Simulation. Figure 23 shows the axis-wise plot of raw AprilTag data (red) and the particle filter output (blue). It shows the filter is filtering the noise and improving the overall performance.

## 5. Conclusions

Fiducial markers are a low-cost solution for getting accurate ground truth measurements in applications, especially in robotics. Among all state of the art fiducial markers, AprilTag is the most commonly used fiducial marker by the researchers. Fast and sturdy tag detection techniques, stronger digital coding for an embedded marker, robust against different lighting conditions, lens distortion and small occlusion are the main features that make AprilTag unique from other fiducial markers. However, researchers have experienced that AprilTag lacks the required precision and accuracy for delicate tasks. Hence, researchers have used a different combination of sensors along the AprilTag to improve its accuracy. In this paper, we have empirically analyzed AprilTag with the identification of shortcomings causing inaccuracies. With the help of extensive experiments and analysis, we have analytically identified that the primary source of error is the yaw angle variation of the viewing camera, which has not been compensated in the current AprilTag implementation. Other inaccuracy sources include distance and viewing angle of the camera to tag.

Besides, based upon the identified shortcomings, three improvement approaches have been proposed to further improve the accuracy and precision of AprilTag with slight execution time trade-off. The first proposed approach includes passive correction of camera yaw angle by using trigonometric corrections to point the camera towards the center of AprilTag. The second approach uses a custom build hardware-based tracking gimbal to align the face of the camera towards the center of tag. Lastly, we have demonstrated how to use the experimental data to build a probabilistic model of the AprilTag sensor using Gaussian Processes (GP) Regression that can be reused as a forward sensor model in many localization-based applications. Also, we have demonstrated that the accuracy and precision of AprilTag increase manifolds if we use the proposed approaches in combination with each other. Comparative results with the Motion Capture (MoCap) system have been shown to best show the improvement proposed.

The suggested enhancement approaches can be used in multiple applications, including robotics and virtual reality (VR). We have experimentally tested the proposed approaches in both indoor and outdoor environments to show the completeness of the proposed probabilistic sensor model. Nonetheless, we have only analyzed horizontal, vertical and yaw axis accuracy reported by the AprilTag, which is sufficient for many ground-based localization applications. However, for more complex operations in 6-DOF environments like aerial robotics, other axes such as height, roll and pitch axis are also principal. Further work needs to be done in that direction.

Moreover, for hard real-time applications, we believe that the proposed custom build yaw-axis gimbal for active correction of camera yaw angle does not move fast enough to match the hard time constraints. The proposed approach can be further improved by using FPGA based implementation for a quick response. Furthermore, a servo motor can also be improved to increase the speed of tracking. Also, the theoretical framework for GP regression in this paper makes some assumptions and simplifications that need further investigation. Another possible open direction of future work might be the inclusion of multiple sensors tag in the probabilistic sensor model to enhance the performance further. Furthermore, by fusing the data of the Inertial Measurement Unit (IMU) while tracking AprilTag can enhance the performance. It may improve the robustness of the robot generated trajectory by filling the gaps when AprilTag is not detected. Nonetheless, there exist multiple directions for extension of this work, which we have attempted to make accessible to the robotics community for reuse in their research [47] and lay the exposition open to critical examination and investigation of the community.

## 6. Code & Dataset

Proposed improved AprilTag Code and datasets can be accessed/downloaded at http://cyphynets.lums.edu.pk/index.php/Apriltag.

## Figures and Tables

**Table 1:** Commonly used different fiducial markers with key features.

Tag Names	Key Features
ARToolkit [8]	Use solid black outline for quick and robust detection.
Multi-ring Marker [9]	Use color rings instead of black marker for more robust detection.
TRIP [10]	Use a 2D circular mark for location identification.
ARTag [11]	Robustness marker detection against different lightening conditions.
ARToolKitPlus [12]	ARToolKit algorithm has been optimized for embedded devices.
Fourier-Tag [13]	Use robust tag encoding scheme using the phase spectrum of a 1-D signal (gray-scale).
RUNE-Tag [14]	Use perspective properties of circular dots for high accuracy and robustness.
CircularTag [15]	Use circular nature and non-linear optimization to further increase accuracy.
AprilTag [6]	Use stronger digital encoding, robust against different lighting conditions and occlusions.

**Table 2:** Measurement from Motion Capture (MoCap).

Nominal Reference Points		Motion Capture (MoCap) Readings				
$x_{r}$(cm)	$y_{r}$(cm)	N	$\mu_{\bar{x}}$(cm)	$\mu_{\bar{y}}$(cm)	$\sigma_{\bar{x}}^{2}$(cm${}^{2}$)	$\sigma_{\bar{y}}^{2}$(cm${}^{2}$)
0	30	217	0.7062	30.3437	0.003110	0.003120
6	30	195	6.9367	29.5193	0.004890	0.000590
−6	30	193	−3.9230	30.1210	0.045800	0.003080
0	50	204	2.9902	50.0462	0.09500	0.014700
15	50	202	17.7118	49.6042	0.009490	0.002650
−15	50	205	−12.0469	50.8171	0.000168	0.000242
0	70	217	3.2683	70.0810	0.002380	0.000267
20	70	199	23.3681	69.4768	0.000210	0.000197
−20	70	212	−16.8097	70.9505	0.037100	0.001090

**Table 3:** Measurement stats with camera *z*-axis pointed towards the center of AprilTag.

Nominal Reference Points		Ground Truth (MoCap)		AprilTag Readings				
$x_{r}$(cm)	$y_{r}$(cm)	$\bar{x}$(cm)	$\bar{y}$(cm)	N	$\mu_{\bar{x}}$(cm)	$\mu_{\bar{y}}$(cm)	$\sigma_{\bar{x}}^{2}$(cm${}^{2}$)	$\sigma_{\bar{y}}^{2}$(cm${}^{2}$)
0	30	0.0166	30.020	110	−0.2420	30.1510	0.002000	0.000170
6	30	7.0161	30.010	115	7.0161	29.8573	0.000040	0.000040
−6	30	−5.9798	29.92	80	−5.9798	30.4293	0.000035	0.002080
0	50	0.102	49.960	107	0.7571	50.0819	0.000930	0.000002
15	50	14.952	50.69	113	16.9141	49.4206	0.000090	0.000030
−15	50	−14.98	49.90	103	−16.9185	49.4014	0.000090	0.000034
0	70	0.003	70.05	134	1.3080	70.0264	0.007390	0.000014
20	70	20.06	70.06	144	22.3574	69.0718	0.000310	0.000092
−20	70	−20.01	70.02	151	−21.7560	69.5979	0.000240	0.000063

**Table 4:** Measurement stats with AprilTag center does not lie on the *z*-axis of the camera (changing camera yaw angle ‘ϕ’).

Nominal Reference Points		Ground Truth (MoCap)		AprilTag Readings				
$x_{r}$(cm)	$y_{r}$(cm)	$\bar{x}$(cm)	$\bar{y}$(cm)	N	$\mu_{\bar{x}}$(cm)	$\mu_{\bar{y}}$(cm)	$\sigma_{\bar{x}}^{2}$(cm${}^{2}$)	$\sigma_{\bar{y}}^{2}$(cm${}^{2}$)
0	30	0.7062	30.3437	152	−1.1162	30.1501	9.47	0.16
6	30	6.9367	29.5193	182	5.4598	29.4149	14.0	0.56
−6	30	−3.9230	30.1210	162	−6.4092	29.6787	14.0	1.07
0	50	2.9902	50.0462	133	−1.3508	49.1515	76.0	1.51
15	50	17.7118	49.6042	144	13.8889	48.8854	84.0	6.87
−15	50	−12.0469	50.8171	186	−15.5709	48.3046	57.0	7.43
0	70	3.2683	70.0810	163	−0.0285	68.3890	194.0	2.61
20	70	23.3681	69.4768	149	23.4214	67.4216	154.0	14.0
−20	70	−16.8097	70.9505	140	−24.4433	67.4337	112.0	14.0

**Table 5:** (Worst scenario) Measurement stats with fixed camera yaw angle ‘ϕ’ at $110^{\circ }$.

Nominal Reference Points			Ground Truth (MoCap)		AprilTag Readings				
**$x_{r}$(cm)**	**$y_{r}$(cm)**	**ϕ(deg)**	**$\bar{x}$(cm)**	**$\bar{y}$(cm)**	**N**	**$\mu_{\bar{x}}$(cm)**	**$\mu_{\bar{y}}$(cm)**	**$\sigma_{\bar{x}}^{2}$(cm${}^{2})$**	**$\sigma_{\bar{y}}^{2}$(cm${}^{2})$**
0	30	110	0.020	30.001	113	−1.2057	30.5027	0.000230	0.000003
10	30	110	10.07	30.01	154	−0.3694	30.7558	0.021100	0.003230
−10	30	110	−9.67	30.06	178	−0.5791	31.1269	0.000101	0.000021
0	50	110	0.100	50.071	147	−0.8202	50.5234	0.001350	0.000004
15	50	110	14.960	50.100	120	−1.9371	51.8464	0.000210	0.000046
−15	50	110	−14.91	49.97	117	−2.2290	52.4599	0.000165	0.000034
0	70	110	0.03	70.01	184	1.1841	70.4607	0.013600	0.000015
20	70	110	20.10	69.98	102	0.3113	71.4872	0.000750	0.000039
−20	70	110	−20.08	70.05	128	−1.8619	72.1167	0.000425	0.000018

**Table 6:** Table showing measurement stats after applying Soft Yaw Axis Correction (SYAC) on raw AprilTag data.

Nominal Reference Points		Ground Truth (MoCap)		AprilTag Readings				
**$x_{r}$(cm)**	**$y_{r}$(cm)**	**$\bar{x}$(cm)**	**$\bar{y}$(cm)**	**N**	**$\mu_{\bar{x}}$(cm)**	**$\mu_{\bar{y}}$(cm)**	**$\sigma_{\bar{x}}^{2}$(cm${}^{2})$**	**$\sigma_{\bar{y}}^{2}$(cm${}^{2})$**
0	30	0.7062	30.3437	113	−0.5813	30.6166	0.31	0.11
6	30	6.9367	29.5193	154	6.4232	29.7444	0.27	0.31
−6	30	−3.9230	30.1210	178	−6.2826	30.5416	0.20	0.44
0	50	2.9902	50.0462	147	0.1930	51.3185	3.06	0.91
15	50	17.7118	49.6042	120	17.3150	49.5959	3.32	3.37
−15	50	−12.0469	50.8171	117	−16.2733	50.1972	1.12	5.04
0	70	3.2683	70.0810	184	1.8551	71.8400	30.0	3.37
20	70	23.3681	69.4768	102	24.8826	70.5867	12.0	13.0
−20	70	−16.8097	70.9505	128	−26.4522	69.1570	10.0	19.0

**Table 7:** Use of yaw axis gimbal on raw AprilTag system).

Nominal Reference Points		Ground Truth (MoCap)		AprilTag Readings				
**$x_{r}$(cm)**	**$y_{r}$(cm)**	**$\bar{x}$(cm)**	**$\bar{y}$(cm)**	**N**	**$\mu_{\bar{x}}$(cm)**	**$\mu_{\bar{y}}$(cm)**	**$\sigma_{\bar{x}}^{2}$(cm${}^{2}$)**	**$\sigma_{\bar{y}}^{2}$(cm${}^{2})$**
0	30	0.642	31.006	156	−2.7569	30.3124	3.39	0.07
6	30	6.71	29.820	144	5.8448	30.0716	1.65	0.08
−6	30	−5.89	29.851	126	−6.6391	29.8201	2.99	0.19
0	50	2.017	50.02	124	−2.9412	49.8801	6.92	0.08
15	50	16.90	51.13	115	15.9652	49.6746	4.71	0.49
−15	50	−14.42	49.63	144	−15.6127	49.4842	4.16	0.49
0	70	−2.10	68.90	149	−5.5017	70.0076	7.35	0.11
20	70	22.70	71.16	152	21.0146	69.8425	5.84	0.58
−20	70	−21.10	71.23	138	−21.7858	69.7444	5.92	0.56

**Table 8:** Use of yaw axis gimbal with consistent frames (SYAC).

Nominal Reference Points		Ground Truth (MoCap)		AprilTag Readings				
$x_{r}$ **(cm)**	**$y_{r}$(cm)**	**$\bar{x}$(cm)**	**$\bar{y}$(cm)**	**N**	**$\mu_{\bar{x}}$(cm)**	**$\mu_{\bar{y}}$(cm)**	**$\sigma_{\bar{x}}^{2}$(cm${}^{2})$**	**$\sigma_{\bar{y}}^{2}$(cm${}^{2})$**
0	30	0.642	31.006	156	−1.4932	30.6753	0.06	0.08
6	30	6.71	29.820	144	6.7595	29.7747	0.01	0.03
−6	30	−5.89	29.851	126	−5.5643	30.4668	0.05	0.17
0	50	2.017	50.02	124	−2.1262	50.1620	0.19	0.13
15	50	16.90	51.13	115	16.8541	49.2115	0.19	0.43
−15	50	−14.42	49.63	144	−14.8491	50.1751	0.10	0.42
0	70	−2.10	68.90	149	−4.3831	70.2868	1.63	0.05
20	70	22.70	71.16	152	21.9349	69.3702	0.71	0.43
−20	70	−21.10	71.23	138	−20.8403	70.5489	0.29	0.60

**Table 9:** Comparison of AprilTag against various proposed approaches.

Ground-Truth (cm)		Error in Mean(μ) Using Different Approaches for AprilTag. (cm)									
**Motion Capture System (MoCap)**		**Raw AprilTag Readings (Camera Pointing Towards Tag’s Center)**		**Raw AprilTag Readings (Camera Pointing Away from Tag’s Center)**		**Applying Soft Yaw Angle Correction (SYAC) on Raw AprilTag Readings**		**Applying Active Correction with Yaw Axis Gimbal on Raw AprilTag Readings**		**Applying (SYAC + Active Yaw Axis Gimbal Correction) on Raw AprilTag Readings**	
$\bar{x}$	$\bar{y}$	$|\bar{x}-\mu_{\bar{x}}|$	$|\bar{y}-\mu_{\bar{y}}|$	$|\bar{x}-\mu_{\bar{x}}|$	$|\bar{y}-\mu_{\bar{y}}|$	$|\bar{x}-\mu_{\bar{x}}|$	$|\bar{y}-\mu_{\bar{y}}|$	$|\bar{x}-\mu_{\bar{x}}|$	$|\bar{y}-\mu_{\bar{y}}|$	$|\bar{x}-\mu_{\bar{x}}|$	$|\bar{y}-\mu_{\bar{y}}|$
0.642	31.00	0.884	0.855	1.758	0.855	1.223	0.389	3.3989	0.693	2.135	0.330
6.71	29.82	0.3061	0.037	1.250	0.405	0.286	0.075	0.8652	0.251	0.049	0.045
−5.89	29.85	0.0898	0.578	0.519	0.172	0.392	0.690	0.7491	0.030	0.325	0.615
2.017	50.02	1.2599	0.0618	3.367	0.868	1.824	1.298	4.958	0.139	4.143	0.141
16.90	51.13	0.0141	1.709	3.011	2.244	0.415	1.534	0.934	1.455	0.045	1.918
−14.42	49.63	2.4985	0.228	1.150	1.325	1.853	0.567	1.192	0.145	0.429	0.545
−2.10	68.90	3.408	1.126	2.071	0.511	3.955	2.940	3.401	1.107	2.283	1.386
22.70	71.16	0.3426	2.088	0.721	3.738	2.182	0.573	1.685	1.317	0.765	1.789
−21.10	71.23	0.6559	0.432	3.343	2.596	5.352	0.873	0.685	0.285	0.259	0.518

**Table 10:** Average execution time for different approaches.

Approaches	Average Execution Time Per Input Image
Raw AprilTag implementation	90 ms
Passive yaw axis correction (SYAC)	130 ms
Active Correction with yaw axis gimbal	125 ms
(Passive yaw axis + Active gimbal) correction	255 ms

**Table 11:** GP predicted distributions at unseen points against α=0.01,β=20000

Unknown Points	Predictive Distribution		Experimental Distribution	
**($\bar{x},\bar{y},\bar{\theta }$(cm,deg))**	$\mu_{f_{*}^{\mathrm{test}}}$ (cm)	$\sigma_{f_{*}^{\mathrm{test}}}^{2}$ (cm${}^{2})$	$\mu_{x_{*}}$ (cm,deg)	$\sigma_{x_{*}}^{2}$ (cm${}^{2})$
(0,30,90)	(0.4,30.9,95.3)	($3.9\times 10^{-8},2.2\times 10^{-6},2.6\times 10^{-6}$)	(0.1,30.6,89.77)	($6.1\times 10^{-4},1.4\times 10^{-4},1.4\times 10^{-8}$)
(10,30,100)	(11.9,30.9,92.2)	($1.3\times 10^{-5},2.2\times 10^{-6},8.6\times 10^{-6}$)	(10.4,29.5,70.57)	($3.7\times 10^{-5},4.7\times 10^{-5},4.7\times 10^{-9}$)
(0,50,80)	(0.4,49.9,88.56)	($3.9\times 10^{-8},3.0\times 10^{-6},2.2\times 10^{-6}$)	(1.0,51.8,88.84)	($7.5\times 10^{-4},1.0\times 10^{-5},1.0\times 10^{-9}$)
(−15,50,100)	(−16.01,49.9,92.2)	($3.3\times 10^{-5},3.0\times 10^{-6},8.6\times 10^{-6}$)	(−18.8,52.7,109.6)	($1.1\times 10^{-4},1.2\times 10^{-5},1.2\times 10^{-9}$)
(20,70,100)	(20.7,70.01,92.2)	($1.9\times 10^{-4},4.5\times 10^{-6},8.6\times 10^{-6}$)	(22.1,67.0,72.1)	($1.1\times 10^{-4},1.1\times 10^{-4},1.1\times 10^{-8}$)
(−20,70,100)	(−21.3,70.01,92.2)	($4.9\times 10^{-5},4.5\times 10^{-6},8.6\times 10^{-6}$)	(−26.1,78.0,109.8)	($1.8\times 10^{-4},2.4\times 10^{-5},2.4\times 10^{-9}$)

## Abbreviations

The following abbreviations are used in this manuscript:

MAV	Micro Aerial Vehicle
UAV	Unmanned Aerial Vehicle
2D	Two Dimensional
3D	Three Dimensional
DOF	Degree of Freedom
MoCap	Motion Capture System (Vicon MX F-49)
PID	Proportional-Integral-Derivative
GP	Gaussian Processes
InC	Inconsistent
C	Consistent
G+InC	Gimbal with inconsistent
G+Con	Gimbal with consistent
